# Pancreatic Cancer‐Derived Extracellular Vesicles Enriched with miR‐223‐5p Promote Skeletal Muscle Wasting Associated with Cachexia

**DOI:** 10.1002/advs.202504064

**Published:** 2025-07-02

**Authors:** Kangjing Xu, Rongxi Shen, Li Zhang, Xuejin Gao, Xinbo Wang, Changhua Zhang, Xi Chen, Xinying Wang

**Affiliations:** ^1^ Clinical Nutrition Service Center Department of General Surgery Nanjing Jinling Hospital Affiliated Hospital of Medical School Nanjing University Nanjing Jiangsu 210000 China; ^2^ Department of General Surgery Nanjing Jinling Hospital Affiliated Hospital of Medical School Nanjing University Nanjing Jiangsu 210000 China; ^3^ Guangdong Provincial Key Laboratory of Digestive Cancer Research The Seventh Affiliated Hospital of Sun Yat‐sen University Shenzhen Guangdong 518107 China; ^4^ State Key Laboratory of Pharmaceutical Biotechnology School of Life Sciences Nanjing University Nanjing Jiangsu 210023 China

**Keywords:** cancer cachexia, extracellular vesicles, miR‐223‐5p, muscle wasting, pancreatic ductal adenocarcinoma

## Abstract

Pancreatic ductal adenocarcinoma (PDAC) with cachexia‐related muscle wasting as the main manifestation is associated with poor overall survival. Extracellular vesicles (EVs) are key mediators of inter‐organ communication. Here, EVs and EV‐microRNAs (miRNAs) are identified as mediate PDAC‐skeletal muscle communication. EVs are isolated from PDAC patients, mouse models, patients‐derived organoids, and mouse pancreatic cancer cells. Plasma‐derived EVs from PDAC patients or mice are observed to remarkably induced muscle wasting in vitro and in vivo. Depletion of miRNA cargo in these EVs significantly alleviates their detrimental effects on skeletal muscles. Deep RNA sequencing is conducted to profile differentially expressed miRNAs in plasma EVs from patients with or without PDAC. The findings reveal that the expression of miR‐223‐5p expression in PDAC patients’ plasma EVs is negatively associated with the 3‐year overall survival. Mechanistic studies show that miR‐223‐5p contributes to reduced METTL14 transcription by targeting MAFA, associated with decreased m^6^A methylation in skeletal muscles and muscle wasting. This study highlights the absorption of miRNA in PDAC‐derived EVs by skeletal muscles and reveals a previously unrecognized function of PDAC‐derived EV‐miR‐223‐5p in tumor‐muscle inter‐organ communication, offering novel insight into EV‐miR‐223‐5p‐based diagnostic and therapeutic strategies for PDAC patients with sarcopenia upon further validation.

## Graphical abstract

EVs and EV‐microRNAs (miRNAs) mediate PDAC‐skeletal muscle communication. miR‐223‐5p reduces METTL14 transcription by targeting MAFA, which subsequently decreases m^6^A methylation in skeletal muscles, ultimately leading to muscle wasting. This study highlights the absorption of miRNA in PDAC‐derived EVs by skeletal muscles and reveals a previously unrecognized function of PDAC‐derived EV‐miR‐223‐5p in tumor‐muscle inter‐organ communication.

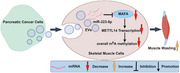

## 1 Introduction

Pancreatic ductal adenocarcinoma (PDAC) has the highest mortality rate of all cancer types, with a 5‐year survival rate of only 8%.^[^ ^1^ ^]^ In ≈15% of newly diagnosed cases where the tumor is completely resectable, most patients ultimately experience local or distant tumor recurrence within two years of surgery.^[^ ^2^, ^3^ ^]^ This reoccurrence is usually associated with the rapid development of cachexia—a systematic metabolic dysfunction characterized by progressive weight loss and muscle wasting, often occurring independently of adipose loss.^[^ ^4^ ^]^ More than 80% of patients with pancreatic cancer suffer from cachexia, a syndrome that is not reversible by conventional nutritional intervention.^[^ ^5^, ^6^ ^]^ The primary feature of cancer cachexia is muscle wasting, driven largely by substantially increased muscle protein breakdown.^[^ ^7^ ^]^ This syndrome significantly deteriorates patients’ quality of life, decreases chemotherapy tolerance, and ultimately accelerates tumor progression in those with pancreatic cancer.^[^ ^8^ ^]^ Despite its profound impact, the biological mechanisms underlying cachexia remain poorly understood, highlighting an urgent need for research into the molecular pathways of cancer cachexia progression to identify viable therapeutic targets.

Tumor cells play a central role in cancer cachexia, producing various bioactive factors that act as mediators to synergistically communicate with skeletal muscles. Numerous factors secreted by tumor tissues—such as transforming growth factor–β and ectodysplasin A—act on skeletal muscles, modulating their metabolic activity.^[^ ^9^, ^10^ ^]^ Additionally, pro‐inflammatory cytokines like interleukin (IL)‐1 and IL‐6 can trigger systemic inflammation and promote muscle atrophy.^[^ ^11^, ^12^ ^]^ However, the mechanisms governing interactions between cancer cells and skeletal muscles in cachexia remain to be elucidated.

In addition to the aforementioned conventional soluble factors, tumor cells also release extracellular vesicles (EVs) as insoluble mediators that facilitate inter‐organ communication and regulate the functions of recipient organs.^[^ ^13^ ^]^ Beyond reprogramming cells within the tumor microenvironment and metastatic niches to promote tumor growth, metastasis, and immune evasion, cancer cell‐derived EVs can also affect distant organs, including skeletal muscle—a tissue not typically colonized by cancer cells.^[^ ^14^, ^15^ ^]^ Increasing evidence demonstrates that EVs from various cancers are integral to the development of cachexia‐associated muscle wasting.^[^ ^16^, ^17^ ^]^ The proteins, lipids, and nucleic acids (DNA, mRNA, microRNA [miRNA], long non‐coding RNA [lncRNA], etc.) within EVs may contribute to cancer cachexia in multiple ways.^[^ ^18^ ^]^ For example, in pancreatic and esophageal cancer, EV‐associated proteins such as HSP70/90 and P4HB serve as tumor‐derived signaling factors that trigger muscle degradation, highlighting the significant role of EVs in muscle loss.^[^ ^19^ ^]^ Importantly, previous studies suggest that miRNAs such as miR‐21a‐5p, miR‐26a, miR‐150‐5p, and miR‐155‐5p may participate in the regulation of skeletal muscle wasting.^[^ ^20^ ^]^ For example, EV‐miR‐21 promotes apoptosis through JNK activation to promote muscle wasting in cancer cachexia.^[^ ^21^ ^]^ EV‐miR‐26a prevents muscle atrophy by inhibiting the transcription factor forkhead box O1 in obstructive kidney disease.^[^ ^22^ ^]^ Cancer‐derived miR‐195a‐5p/miR‐125b‐1‐3p may induce muscle atrophy by targeting Bcl‐2‐mediated apoptosis in colon cancer cachexia.^[^ ^23^ ^]^ However, whether EV‐miRNAs are involved in communication between pancreatic cancer cells and skeletal muscle remains unclear.

In this study, we uncovered novel mechanisms of inter‐organ communication between pancreatic tumor tissue and skeletal muscle, demonstrating that tumor‐derived EVs and their miRNA cargo can be transported to muscle tissue. Through EV‐miRNA sequencing in plasma, we identified EV‐miR‐223‐5p as a key mediator in PDAC‐muscle interactions and determined that the MAF bZIP transcription factor A (MAFA) is a direct target, capable of regulating the methyltransferase‐like 14 (METTL14)‐N6‐methyladenosine (m^6^A) axis in muscle. These results reveal the crucial regulatory role of PDAC‐derived EV‐miR‐223‐5p and METTL14‐mediated m^6^A methylation in muscle wasting, offering potential therapeutic strategies for managing sarcopenia in PDAC patients.

## 2 Results

### 2.1 Pancreatic Cancer Induces Cachexia‐Associated Muscle Wasting

**Figure 1:**
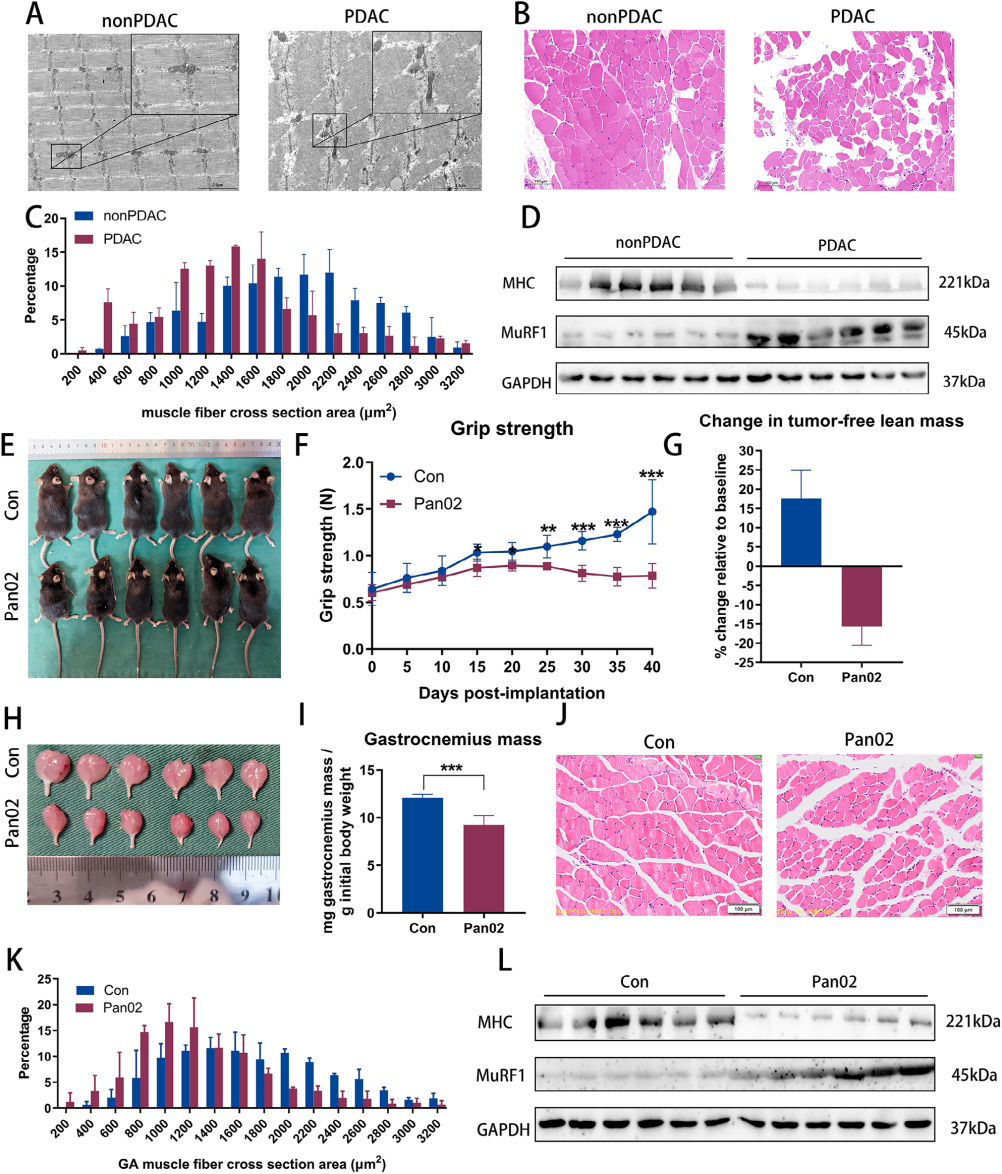
Pancreatic cancer induces cachexia‐associated muscle wasting. A) Representative ultrastructural images of the rectus abdominis muscle from nonPDAC and PDAC patients. Scale bars, 2 µm. B) HE staining of the rectus abdominis muscle from nonPDAC and PDAC patients. Scale bars, 100 µm. C) Quantitative analysis of CSA of muscle fibers in Figure 1B (n = 6). D) Western blot of MHC and MuRF1 protein in rectus abdominis muscle from nonPDAC and PDAC patients. E—I) Mouse images (E), grip strength changes (F), lean mass changes (G), GA muscle images (H), and muscle weight analysis (I) of tumor‐bearing mice (Pan02, n = 6) and non‐tumor‐bearing control (Con, n = 6) mice. J) HE staining of the GA muscle from tumor‐bearing mice and non‐tumor‐bearing control mice. Scale bars, 100 µm. K) Quantitative analysis of CSA areas of muscle fibers in Figure 1J (n = 6). L) Western blot of MHC and MuRF1 protein in the GA muscle of tumor‐bearing mice and non‐tumor‐bearing control mice. Data shown as mean ± SD. ^*^ *P* < 0.05, ^**^ *P* < 0.01, ^***^ *P* < 0.001.

To evaluate the adverse effects of pancreatic cancer on skeletal muscle wasting, we collected rectus abdominis and subcutaneous adipose tissue (SAT) samples from patients with or without PDAC (PDAC group and nonPDAC group). Electron microscopy of rectus abdominis muscles from the nonPDAC group displayed a well‐organized sarcomere structure, with parallel‐aligned myofibrils and properly arranged mitochondria flanking the Z‐lines. In contrast, the muscle ultrastructure in the PDAC group showed various degrees of disorganized tissue, marked by misaligned sarcomeres and Z‐lines as well as swollen mitochondria (**Figure** **1**A). Histological analysis further revealed a significant reduction in myofiber cross‐sectional area (CSA) in the PDAC group (Figure 1B). Most myofibers in the PDAC group exhibited notably smaller CSA and increased interstitial space compared to those in the nonPDAC group (Figure 1C). Consistently, protein analysis showed elevated muscle‐RING‐finger‐1 (MuRF1) and reduced myosin heavy chain (MHC) levels in the PDAC group compared to the nonPDAC group (Figure 1D; Figure S1A, Supporting Information). Hematoxylin and eosin (HE)‐stained SAT also indicated a substantial decrease in adipocyte area in the PDAC group (Figure S1B,C, Supporting Information). Taken together, these results demonstrate that PDAC systematically induces cachexia and muscle loss at the histological, microstructural, and protein levels.

A cachexia model was established by injecting the mouse pancreatic cancer cell line Pan02 into the pancreas tail of C57BL/6J mice to investigate the mechanisms behind PDAC‐ associated muscle wasting in vivo. Mice in the Pan02‐injected group (Pan02 group) exhibited decreased body weight, lean mass, fat mass, food intake, and grip strength compared to non‐tumor‐bearing mice (Con group) (Figure 1E–G; Figure S1D—F, Supporting Information). Consistent with observations from human muscle and adipose tissue samples, muscle wasting and fat depletion associated with cancer cachexia were evident in the Pan02 group (Figure 1H–L; Figure S1H—L, Supporting Information). Histopathological analysis further demonstrated a reduction in myofiber CSA across multiple muscle types, including the tibialis anterior (TA), quadriceps, soleus, and cardiac muscles, in the Pan02 group compared to the Con group (Figure S1G, Supporting Information). A large body of evidence demonstrates that the hypothalamus is a critical driver of cachexia, transducing systemic inflammatory messages stemming from acute and chronic disease processes into a local and paracrine inflammatory response in the central nervous system.^[^ ^24^ ^]^ ELISA analysis showed higher IL‐1β levels in the Pan02 group than in the Con group (Figure S1M, Supporting Information). Collectively, these findings confirm the successful establishment of a PDAC‐induced cachexia model using the Pan02 cell line. This model reproduces the key features of PDAC induced cachexia, including weight loss, anorexia, muscle wasting, and systemic inflammation, thus providing a reliable model for further exploration of the molecular mechanism of PDAC cachexia in the following text.

### 2.2 Pancreatic Cancer‐Derived EVs Induce Skeletal Muscle Wasting

**Figure 2:**
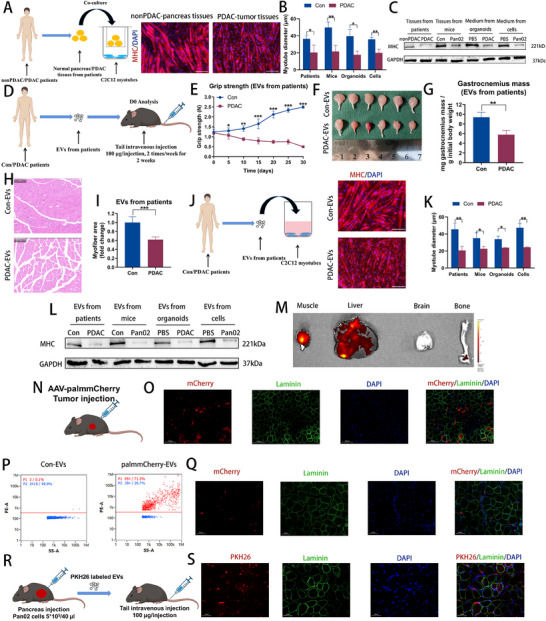
Pancreatic cancer‐derived EVs induce skeletal muscle wasting. A) Left: Experimental scheme: normal pancreas tissues of nonPDAC patients and pancreatic tumor tissues of PDAC patients were collected in sterile procedures and cocultured with C2C12 myoblasts. Right: The representative images of immunofluorescence staining for MHC in C2C12 myoblasts. Scale bars, 100 µm. B) The quantitative analysis of myotube diameters of C2C12 cocultured with pancreas tissues of patients and mice, and CM of organoids and Pan02 cells (n = 3). C) Western blot of MHC protein in C2C12 myoblasts cocultured with pancreas tissues of patients and mice, and CM of organoids and Pan02 cells. D) Experimental scheme: EVs isolated from the plasma of patients with PDAC (PDAC‐EVs) and healthy individuals (Con‐EVs) were injected to mice intravenously for 2 weeks. E—G) Grip strength changes (E), GA muscle images (F), and muscle weight analysis (G) of mice injected with EVs from plasma of control patients (Con, n = 6) and PDAC patients (PDAC, n = 6). H) HE staining of the GA muscle from mice injected with EVs from the plasma of control patients and PDAC patients. Scale bars, 100 µm. I) Quantification of the myofiber CSA in Figure 2H (n = 6). J) Left: Experimental scheme: EVs isolated from the plasma of patients with PDAC (PDAC‐EVs) and healthy individuals (Con‐EVs) were cocultured with C2C12 myoblasts. Right: The representative images of immunofluorescence staining for MHC in C2C12 myoblasts. Scale bars, 100 µm. K) The quantitative analysis of myotube diameters of C2C12 treated with EVs extracted from patients, mice, organoids, and Pan02 cells (n = 3). L) Western blot of MHC protein in C2C12 myoblasts cocultured with EVs of patients, mice, organoids, and Pan02 cells. M) Fluorescence imaging of mouse muscle, liver, bone, and brain tissues after mice were injected with PKH26‐stained EVs from Pan02 cells. N) Experimental scheme: Pan02 mice were injected with AAV‐palmmCherry directly into their tumors. O) Representative immunofluorescent images of mCherry and Laminin in the muscle of AAV‐palmmCherry‐tumor‐injected mice. Scale bars, 100 µm. P) Dot‐plots of EVs from mice injected with AAV‐palmmCherry in their tumors and EVs from mice not injected with AAV‐palmmCherry in their tumors using nano‐flow cytometry. Q) Representative immunofluorescent images of mCherry and Laminin in the muscle of AAV‐palmmCherry‐EVs‐injected mice. Scale bars, 50 µm. R) Experimental scheme: Mice were injected with PKH26‐labeled EVs extracted from tumor‐bearing mice. S) Representative immunofluorescent images of PKH26 and Laminin in the muscle of PKH26‐labeled‐EVs‐injected mice. Scale bars, 50 µm. Data shown as mean ± SD. ^*^ *P* < 0.05, ^**^ *P* < 0.01, ^***^ *P* < 0.001.

To further investigate the role of tumor tissue in PDAC‐induced muscle wasting in vitro, we cocultured C2C12 myotubes with the pancreatic tumor tissues from patients and mice. **Figures** **2**A and S2A (Supporting Information) show that PDAC tumors caused a notable reduction in C2C12 myotube diameter. Similarly, C2C12 myotubes treated with culture medium (CM) from human‐derived PDAC organoids and Pan02 cells appeared thinner than those in the Con group (Figure 2B; Figure S2B–D, Supporting Information). Western blot analysis further revealed a loss of MHC in the PDAC group compared to the Con group (Figure 2C; Figure S2E, Supporting Information). Collectively, these results support the hypothesis that PDAC tumor tissue exerts detrimental effects on muscle, contributing to muscle wasting.

To investigate whether EVs derived from tumors contribute to muscle pathology in PDAC, we administered equivalent amounts of EVs isolated from the plasma of PDAC patients (PDAC‐EVs) and healthy individuals (Con‐EVs) to mice intravenously for 2 weeks (Figure 2D). Prior to injection, EVs were characterized using a combination of TEM, NanoSight particle analysis, and immunoblotting for EV markers such as CD63, CD9, TSG101, and calnexin, confirming that these EVs were enriched with exosomes (Figure S2F—H, Supporting Information). Following EV treatment, mice receiving PDAC‐EVs displayed reduced grip strength, decreased muscle mass, and a significant reduction in muscle CSA compared to Con‐EVs‐treated mice (Figure 2D–I). Notably, mice treated with EVs from plasma of tumor‐bearing mice (Pan02 mice‐EVs), CM of PDAC‐derived organoids (PDAC‐EVs), and CM of Pan02 cells (Pan02‐EVs) exhibited detrimental effects on grip strength and skeletal muscle mass compared to those treated with control EVs (Figure S3, Supporting Information). Additionally, treatment of C2C12 myotubes with these EVs for 48 h resulted in significant myotube atrophy (Figure 2J; Figure S4A—C, Supporting Information), as evidenced by reduced myotube diameter and decreased MHC protein expression compared to Con groups (Figure 2K,L; Figure S4D, Supporting Information). To further explore the role of EVs in cachexia‐associated muscle pathology in vivo, we administered GW4869, an EV secretion inhibitor, intraperitoneally to tumor‐bearing mice for 2 weeks (Figure S4E, Supporting Information). GW4869 treatment substantially mitigated grip strength impairment and muscle mass loss, without changing tumor growth, compared to vehicle treatment (Figure S4F—K, Supporting Information). Additionally, GW4869 inhibited the pro‐atrophic effect of Pan02 cells, as shown by increased myotube diameter in the GW4869‐treated group compared to the Con group (Figure S4L, Supporting Information). These findings indicate that pancreatic cancer‐derived EVs induce muscle wasting both in vitro and in vivo.

To assess the targeting efficiency of EVs to muscle tissues, we intravenously injected PKH26‐labeled EVs derived from Pan02 cells into recipient mice. In vivo imaging revealed that these EVs were highly enriched in mouse muscle tissue compared to their accumulation in the brain and bone (Figure 2M). Palmitoylation (palm) facilitates the attachment of proteins to cellular membranes through the formation of a thioester bond between the cysteine sulfhydryl groups and palmitic acid, a fatty acid. AAV‐palmmCherry is a palm signal genetically fused to the N‐terminus of mCherry,^[^ ^25^ ^]^ which can be injected into the pancreatic tumor to label EVs generated from tumors with mCherry. Therefore, EVs can be labeled from their production in tumors in vivo, enabling the tracing of their origin and tracking of their movement. In Figure 2O, mice that received AAV‐palmmCherry injections directly into their tumors displayed strong fluorescent signals in their skeletal muscles. We could speculate that the fluorescence in muscles was likely attributed to EVs secreted by tumors. However, fluorescence could also be caused by AAV leakage or other reasons, therefore, it cannot be confirmed with certainty that EVs secreted by tumors transmit fluorescence. To further confirm that the fluorescence in muscles was actually caused by EVs secreted by tumors, we isolated EVs from mice that had been injected with AAV‐palmmCherry in their tumors (palmmCherry‐EVs). EVs from mice not injected with AAV were used as the control group (Con‐EVs) for nano‐flow cytometry detection (Figure 2P). The results showed that palmmCherry‐EVs were labeled with mCherry, confirming that EVs secreted by pancreatic tumors entered the circulatory system. The combined results of Figure 2O,P confirmed that tumors indeed secreted EVs, entering the bloodstream, and reaching muscle tissue. Since we found that tumors can secrete EVs to reach the bloodstream, we further injected these palmmCherry‐EVs into the tail vein of another recipient mice to further verify the above conclusion. We still observed fluorescent signals in muscle tissue, which further confirmed the muscle targeting of EVs secreted by tumors (Figure 2Q). In vivo immunofluorescent microscopy further demonstrated that mice injected with PKH26‐labeled EVs from tumor‐bearing mice showed a marked fluorescent signal in their muscles (Figure 2R,S). In vitro experiments corroborated that myotubes internalized PKH26‐labeled EVs from Pan02 cells after 12 h of incubation (Figure S4M, Supporting Information). Collectively, these data imply that pancreatic cancer‐derived EVs effectively target muscle tissue.

### 2.3 MicroRNAs in EVs Derived from Pancreatic Cancer Mediate Muscle Wasting

**Figure 3:**
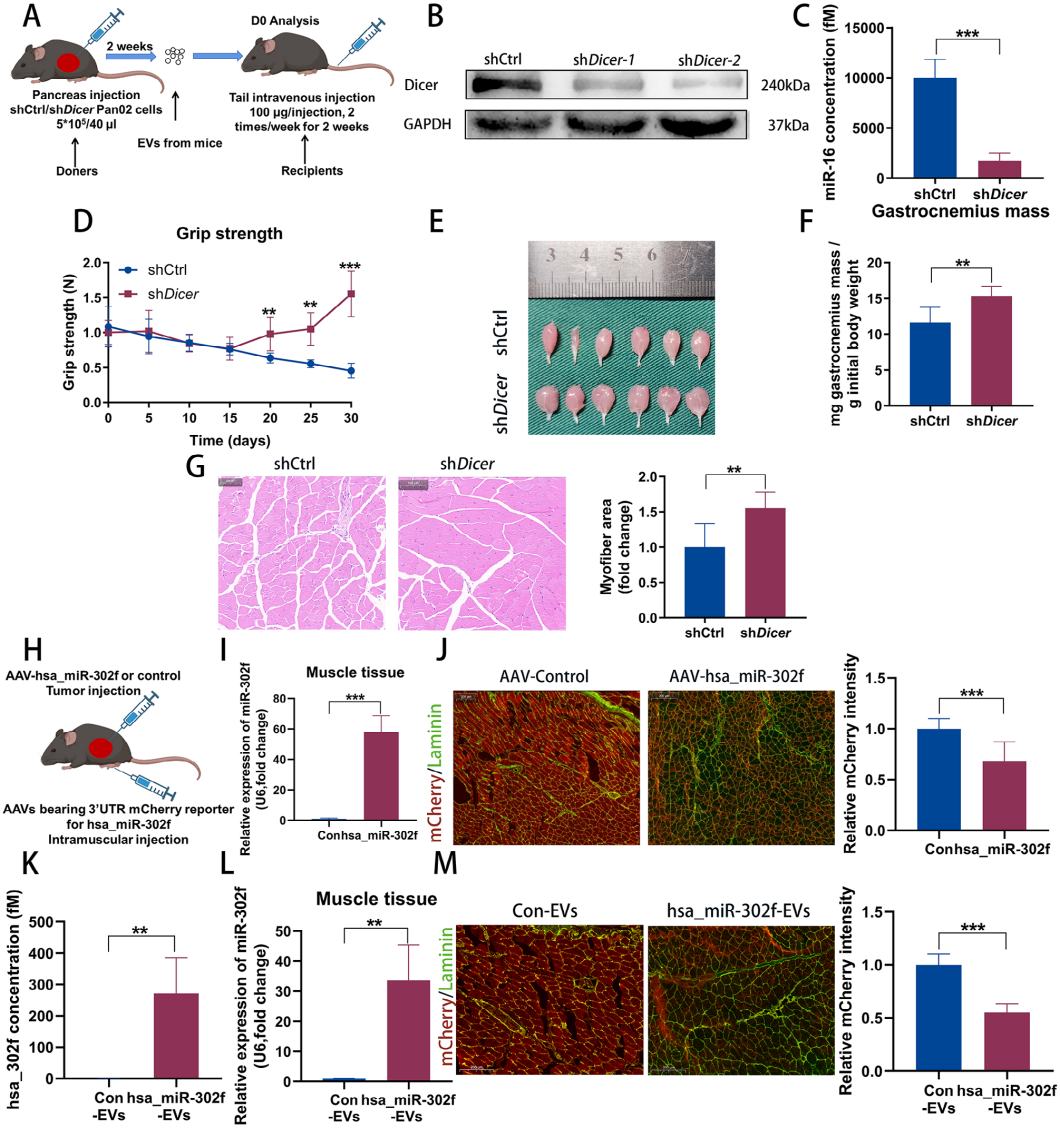
MicroRNAs in EVs derived from pancreatic cancer mediate muscle wasting. A) Experimental scheme: mice were orthotopically implanted with shCtrl and sh*Dicer* Pan02 cells. B) Western blot of Dicer protein in shCtrl and sh*Dicer* Pan02 cells. C) Relative levels of miR‐16 (as an endogenous control for miRNA quantification) of EVs extracted from shCtrl (shCtrl, n = 6) and sh*Dicer* (sh*Dicer*, n = 6) mice. D,E) Grip strength changes (D), GA muscle images (E), and muscle weight analysis F) of mice injected with EVs extracted from shCtrl (shCtrl, n = 6) and sh*Dicer* (sh*Dicer*, n = 6) mice. G) Left: HE staining of the GA muscle from C57BL/6J mice injected with EVs extracted from shCtrl and sh*Dicer* mice. Scale bars, 100 µm. Right: Quantification of the myofiber CSA (n = 6). H) Experimental scheme: AAVs carrying the 3′UTR mCherry reporter for hsa_miR‐302f were administered into the GA muscle of mice. Three days later, AAV‐hsa_miR‐302f or AAV‐Control were injected into the pancreatic tumors of the mice. I) hsa_miR‐302f levels in muscles 2 weeks after AAV‐hsa_miR‐302f or AAV‐Control injection (n = 6). J) Left: Representative immunofluorescent images of mCherry in muscles. Right: Quantitation of mCherry intensity (n = 6). Scale bars, 200 µm. K) AAVs expressing hsa_miR‐302f (AAV‐hsa_miR‐302f) or its control (AAV‐Control) were injected into the tumor tissue of the mice. qRT–PCR analysis of the miR‐302f levels in EVs extracted from AAV‐hsa_miR‐302f injected mice (hsa_miR‐302f‐EVs) or AAV‐Control injected mice (Con‐EVs) (n = 6). L) Two weeks after hsa_miR‐302f‐EVs or Con‐EVs injection, the hsa_miR‐302f levels in the muscles were analyzed via qRT–PCR (n = 6). M) Left: Representative immunofluorescent images of mCherry in muscles. Right: quantitation of mCherry intensity (n = 6). Scale bars, 200 µm. Data shown as mean ± SD. ^*^ *P* < 0.05, ^**^ *P* < 0.01, ^***^ *P* < 0.001.

miRNAs encapsulated within EVs are known to play a critical role in regulating the tissues that receive them.^[^ ^26^ ^]^ To investigate whether miRNAs contribute to EV‐mediated muscle wasting in pancreatic cancer, we orthotopically implanted Pan02 cells with Dicer knockdown (shDicer), an enzyme essential for miRNA processing, into mice (**Figure** **3**A). Dicer knockdown in Pan02 cells effectively suppressed the expression of Dicer in cells (Figure 3B; Figure S5A, Supporting Information). Figure S5B,C (Supporting Information) showed that shDicer tumor‐bearing mice exhibit partial improvement in grip strength and GA muscle mass compared to shCtrl mice. We extracted EVs from these mice and found decreased levels of miRNAs in EVs derived from tumor‐bearing mice (Figure 3C; Figure S5D, Supporting Information). Subsequently, EVs from shCtrl mice (shCtrl‐EVs) or shDicer mice (shDicer‐EVs) were intravenously administered to recipient mice. Mice receiving shDicer‐EVs showed significant improvements in grip strength and a reduction in muscle mass loss (Figure 3D–G). Furthermore, C2C12 myotubes were treated with EVs derived from shCtrl or shDicer Pan02 cells for 48 h. Myotubes treated with EVs from shDicer Pan02 cells were thicker than those in the shCtrl group, suggesting that miRNA depletion in EVs may help mitigate cancer‐induced muscle loss (Figure S5E,F, Supporting Information). These findings indicate that the muscle‐wasting effects of pancreatic cancer‐derived EVs are reduced following the inhibition of Dicer.

Although we have confirmed that PDAC‐derived EVs mediate skeletal muscle wasting through EV‐miRNA, there is still a lack of direct evidence to determine whether their molecular mechanisms depend on miRNA binding directly to the target gene 3′UTR in muscle tissue. In order to unequivocally demonstrate inter‐tissue miRNA regulation, we must eliminate the interference of endogenous miRNAs in mice, such as miRNAs secreted by muscle tissue itself. Thus, AAV‐hsa_miR‐302f and AAV‐3′UTR‐mCherry were applied. It is worth noting that hsa_miR‐302f is a human‐specific miRNA that lacks a mouse homolog,^[^ ^27^ ^]^ making it an ideal molecular tool for eliminating endogenous miRNA interference and accurately analyzing the cross‐tissue regulatory function of tumor‐derived miRNAs in mouse models.^[^ ^13^ ^]^ Based on this, AAV‐3′UTR‐mCherry, carrying the 3′UTR sequence specifically complementary to hsa_miR‐302f, combined with AAV‐hsa_miR‐302f could show the target binding of tumor‐derived miRNA through mCherry fluorescence to clarify the cross‐tissue regulatory ability in mice. AAV‐3′UTR‐mCherry was administered into the GA muscle of mice. Three days following the initial injection, AAVs expressing either hsa_miR‐302f (AAV‐hsa_miR‐302f) or a control sequence (AAV‐Control) were injected into the pancreatic tumors of the mice (Figure 3H). Two weeks post‐injection, mice that received AAV‐hsa_miR‐302f exhibited elevated levels of hsa_miR‐302f and a significant decrease in muscle fluorescence intensity compared to control mice (Figure 3I,J). Additionally, we extracted EVs from mice injected with AAV‐hsa_miR‐302f (hsa_miR‐302f‐EVs) and AAV‐control (Control‐EVs). These EVs were then administered to recipient mice that had been previously injected with AAV expressing an mCherry reporter containing the 3′UTR for hsa_miR‐302f. The hsa_miR‐302f‐EVs exhibited higher levels of hsa_miR‐302f compared to the Control‐EVs (Figure 3K). Two weeks following EV administration, mice treated with hsa_miR‐302f‐EVs had a significant increase in hsa_miR‐302f levels and a reduction in mCherry intensity in muscle tissue compared to control mice (Figure 3L,M). These findings confirm the critical role of miRNAs in PDAC‐derived EVs in regulating skeletal muscle loss.

### 2.4 miR‐223‐5p is Enriched in Plasma EVs of Patients with Cachexia and Functions as a Biomarker for Sarcopenia and Survival in Pancreatic Cancer

**Figure 4:**
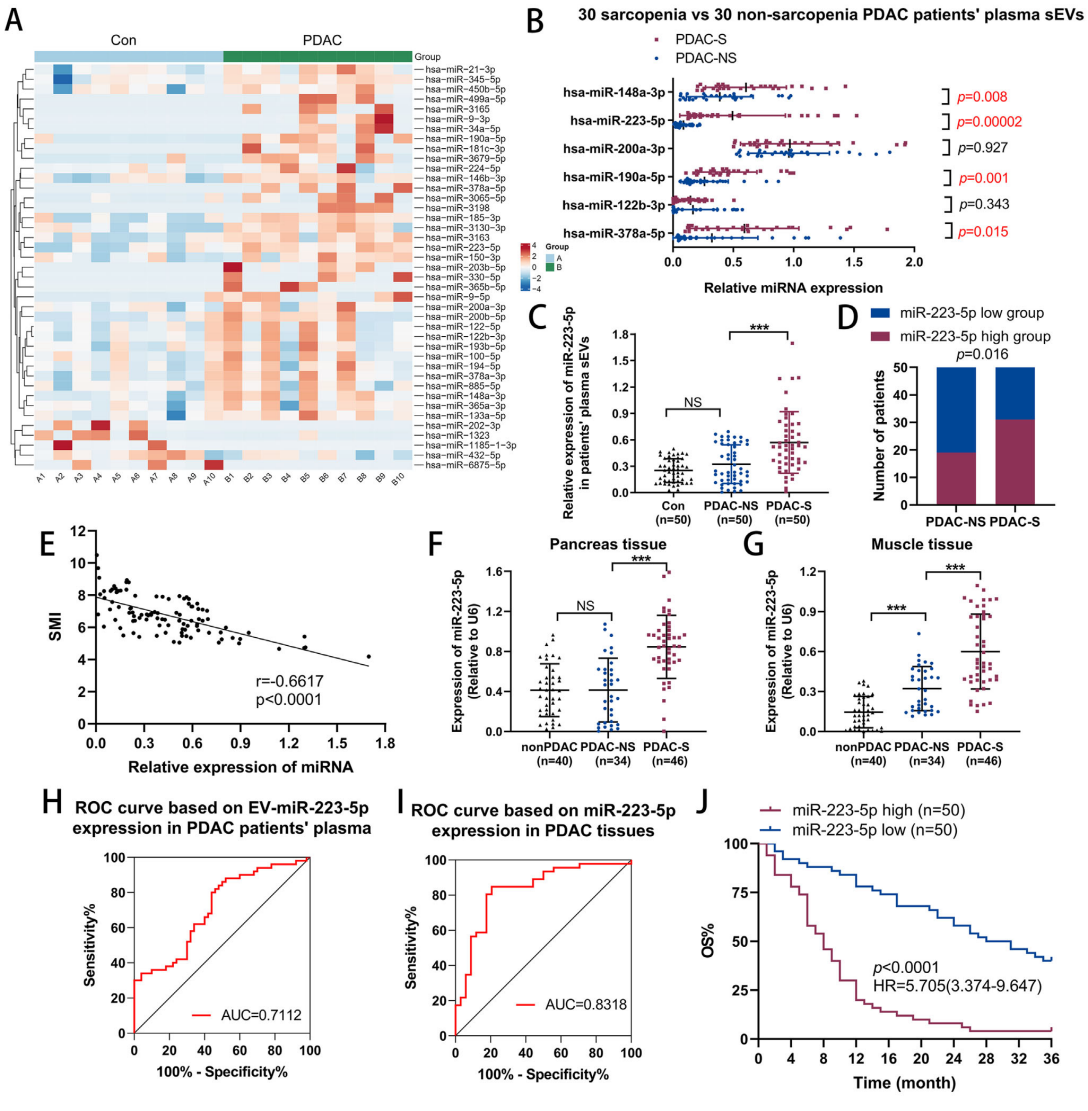
miR‐223‐5p is enriched in plasma EVs of patients with cachexia and functions as a biomarker for sarcopenia and survival in pancreatic cancer. A) The heatmaps of EV‐miRNAs in the plasma of control people (n = 10) as compared to PDAC patients (n = 10). B) The top six EV‐miRNAs based on *P* value were further analyzed in the plasma EVs of 30 sarcopenia PDAC patients (n = 30) and 30 non‐sarcopenia PDAC patients (n = 30) by qRT‐PCR. C) EV‐miR‐223‐5p expression was detected by qRT‐PCR in the plasma of 50 control people, 50 non‐sarcopenia PDAC patients, and 50 sarcopenia PDAC patients. D) The incidence of sarcopenia was compared after patients were divided into high and low groups by the median expression of EV‐miR‐223‐5p in plasma. n = 100. E) Pearson's correlation analysis of the miR‐223‐5p concentration with the SMI in patients with PDAC. n = 100. F,G) Relative miR‐223‐5p expression in pancreas (F) and muscle (G) tissues of 40 nonPDAC patients, 34 non‐sarcopenia PDAC patients, and 40 sarcopenia PDAC patients. H,I) ROC curves based on EV‐miR‐223‐5p expression in the plasma (H) and miR‐223‐5p expression in the tumor tissue samples (I) of PDAC patients. J) Overall survival analysis based on miR‐223‐5p expression in 100 PDAC patients. The median miR‐223‐5p expression was used as the cutoff. Data shown as mean ± SD. ^*^ *P* < 0.05, ^**^P < 0.01, ^***^ *P* < 0.001.

To pinpoint the specific miRNAs in EVs that contribute to muscle wasting, we performed RNA deep sequencing on EVs from 10 PDAC patients and 10 controls (**Figure** **4**A). The top six EV‐miRNAs, ranked by P value, were further validated using qRT‐PCR. Among them, hsa_miR‐223‐5p displayed the most pronounced upregulation in the plasma EVs of 30 PDAC patients with sarcopenia compared to 30 PDAC patients without sarcopenia (Figure 4B). This finding was confirmed in an expanded cohort of 50 healthy volunteers (Con), 50 PDAC patients without sarcopenia (PDAC‐NS), and 50 PDAC patients with sarcopenia (PDAC‐S) (Figure 4C,D). In the overall PDAC group, correlation analysis revealed a significant negative association between miR‐223‐5p levels in plasma EVs and skeletal muscle index (SMI) (Figure 4E). Additionally, miR‐223‐5p expression was significantly elevated in pancreatic tumors and muscle tissues of PDAC patients with sarcopenia (Figure 4F,G). Receiver operating characteristic (ROC) curve analysis suggested that miR‐223‐5p expression in both plasma and tumor tissues had the potential to be used as biomarkers of sarcopenia, with area under the curve values of 0.711 and 0.832, respectively (Figure 4H,I). Moreover, it is well‐established that cachexia‐associated muscle wasting likely arises from the combined effects of multiple miRNAs rather than miR‐223‐5p alone. Therefore, we expanded this analysis to evaluate synergistic diagnostic potential. As shown in Figure 4B, hsa‐miR‐223‐5p, hsa‐miR‐378a‐5p, hsa‐miR‐190a‐5p, and hsa‐miR‐148a‐3p showed significant upregulation in 30 PDAC‐S patients (P < 0.05). We further quantified the expressions of hsa‐miR‐378a‐5p, hsa‐miR‐190a‐5p, and hsa‐miR‐148a‐3p in plasma EVs from an additional 20 PDAC‐NS and 20 PDAC‐S patients (data in Table S2, Supporting Information). Furthermore, we generated an ROC curve using the expression levels of hsa‐miR‐223‐5p, hsa‐miR‐378a‐5p, hsa‐miR‐190a‐5p, and hsa‐miR‐148a‐3p. Strikingly, a combination panel of these miRNAs (miR‐223‐5p/378a‐5p/190a‐5p/148a‐3p) in plasma EVs achieved significantly superior diagnostic accuracy (AUC = 0.832; Figure SS5G, Supporting Information), highlighting the collaborative role of these miRNAs in PDAC‐associated cachexia. Notably, elevated levels of miR‐223‐5p in plasma were associated with poor OS in PDAC patients, as indicated by Kaplan–Meier survival analysis (Figure 4J).

### 2.5 miR‐223‐5p in EVs Derived from Pancreatic Cancer Induces Muscle Wasting

**Figure 5:**
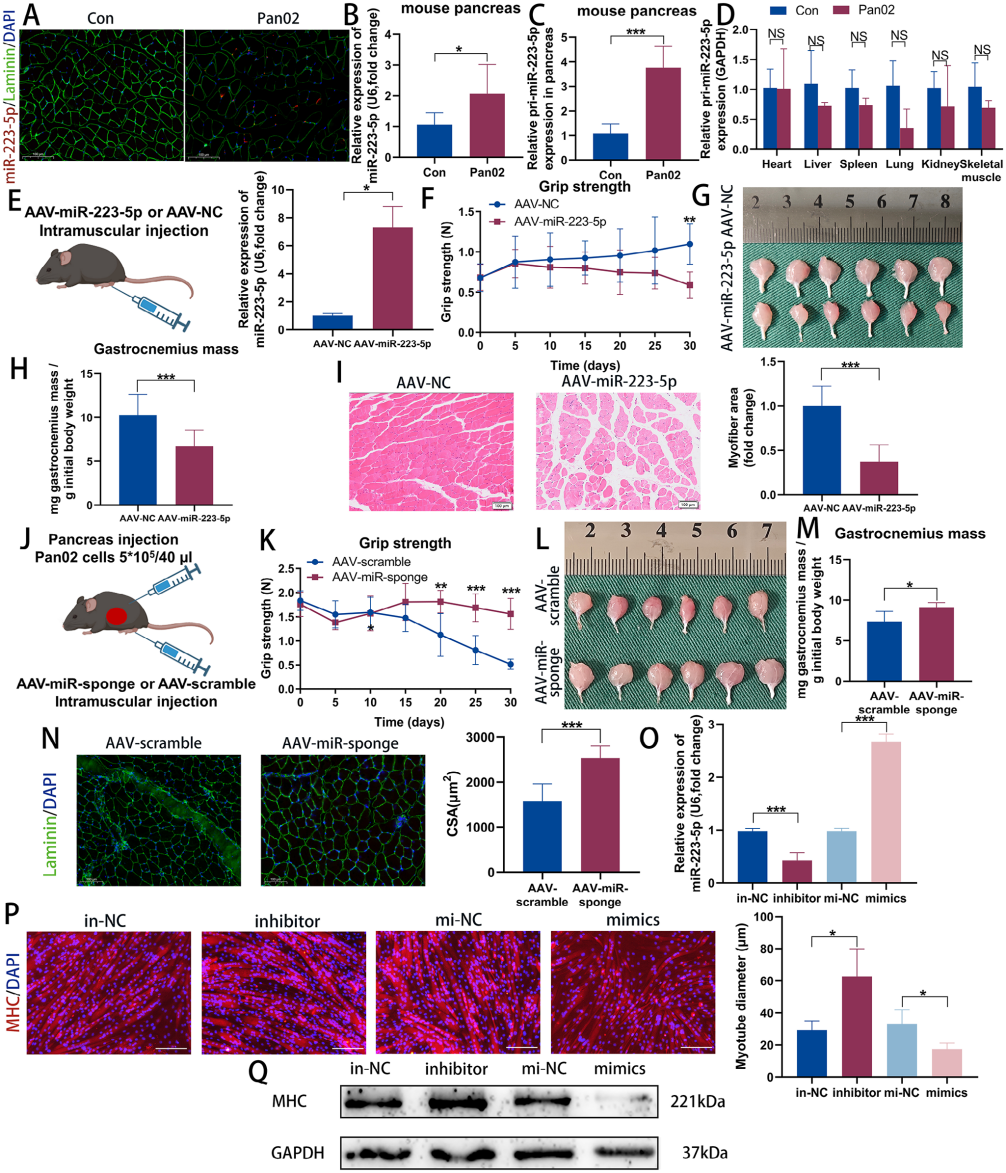
miR‐223‐5p in EVs derived from pancreatic cancer induces muscle wasting. A) Representative immunofluorescent images of FISH analysis of miR‐223‐5p (red) and immunostaining of Laminin (green) in the muscles of con and Pan02 mice. Scale bars, 100 µm. B) miR‐223‐5p level in pancreas tissues of con (n = 6) and Pan02 mice (n = 6). C,D) Primary miR‐223‐5p level in the pancreas (C), lung, kidney, heart, liver, spleen, and skeletal muscle tissues (D) of con (n = 6) and Pan02 mice (n = 6). E) Left: Experimental scheme: AAV‐miR‐223‐5p or AAV‐NC were injected into the GA muscle of the mice. Right: miR‐223‐5p level in muscles of mice 30 days after AAV‐miR‐223‐5p (n = 6) and AAV‐NC (n = 6) injection. F—H) Grip strength changes (F), GA muscle images (G), and muscle weight analysis (H) of mice injected with AAV‐miR‐223‐5p (n = 6) and AAV‐NC (n = 6). I) Left: HE staining of the GA muscle from mice injected with AAV‐miR‐223‐5p and AAV‐NC. Scale bars, 100 µm. Right: Quantification of the myofiber CSA (n = 6). J) Experimental scheme: AAV‐sponge or AAV‐scramble was injected into the GA muscle of mice. Three days after the injection of the AAVs, the mice were injected with Pan02 cells into the tail of the pancreas. K—M) Grip strength changes (K), GA muscle images (L), and muscle weight analysis (M) of Pan02 mice injected with AAV‐miR‐223‐5p‐sponge (n = 6) and AAV‐scramble (n = 6). N) Left: The representative immunofluorescent images of Laminin and DAPI in the muscles of Pan02 mice injected with AAV‐miR‐223‐5p‐sponge and AAV‐scramble. Scale bars, 100 µm. Right: Quantification of the myofiber CSA in Pan02 mice injected with AAV‐miR‐223‐5p‐sponge (n = 6) and AAV‐scramble (n = 6). O) qPCR analysis of miR‐223‐5p level after altering miR‐223‐5p expression in C2C12 myoblast (n = 3). P) Left: The representative images of immunofluorescence staining for MHC in C2C12 myoblasts after altering miR‐223‐5p expression. Scale bars, 100 µm. Right: The quantitative analysis of myotube diameters of C2C12 (n = 3). Q) Western blot of MHC protein in Figure 5P (n = 3). Data shown as mean ± SD. ^*^ *P* < 0.05, ^**^ *P* < 0.01, ^***^ *P* < 0.001.

Following the analysis mentioned above, we further assessed miR‐223‐5p levels in Con and Pan02 mice. Fluorescence in situ hybridization (FISH) of miR‐223‐5p revealed stronger red fluorescence in the Pan02 group, indicating higher miR‐223‐5p expression in the skeletal muscle (**Figure** **5**A). This upregulation of miR‐223‐5p in the pancreatic tumor tissues of Pan02 mice was validated through qPCR analysis (Figure 5B). To investigate the source of the elevated miR‐223‐5p, we measured pri‐miR‐223‐5p levels in various peripheral tissues and the pancreas of mice. Although pri‐miR‐223‐5p was detected across all samples, its levels were significantly higher only in pancreatic tumors and not in the heart, liver, spleen, lungs, kidneys, or skeletal muscles of Pan02 mice, compared to Con mice (Figure 5C,D). This finding suggests that the increased miR‐223‐5p in skeletal muscles likely originates from tumor tissue.

To ascertain whether miR‐223‐5p contributes to muscle loss pathology, we administered AAV expressing miR‐223‐5p and mCherry (AAV‐miR‐223‐5p) or a control (AAV‐NC) into the GA muscle of the mice (Figure 5E; Figure S6A, Supporting Information). Mice treated with AAV‐miR‐223‐5p exhibited decreased grip strength and increased muscle loss compared to control mice (Figure 5F–I). Similarly, the AAV‐9 vector expressing a miR‐223‐5p sponge (AAV‐sponge) or its control (AAV‐scramble) was injected into the GA muscle of mice, followed by the injection of Pan02 cells into the pancreas tail 3 days later (Figure 5J). Mice treated with AAV‐sponge exhibited improved grip strength and reduced muscle loss compared to control mice (Figure 5K–N). To further investigate the role of miR‐223‐5p in muscle wasting in vitro, C2C12 myoblasts were transfected with an miR‐223‐5p inhibitor, a negative control (in‐NC), miR‐223‐5p mimics, or a mimic control (mi‐NC) with 80% transfection efficiency, and subsequently induced to differentiate. Results indicated that miR‐223‐5p expression in myotubes was significantly decreased when treated with the miR‐223‐5p inhibitor, whereas it was notably increased when treated with the miR‐223‐5p mimics (Figure 5O). Immunofluorescence staining and Western blot analysis showed that myoblasts exhibited significantly larger diameters and higher MHC protein levels after transfection with the miR‐223‐5p inhibitor. Conversely, myotube diameter and MHC protein expression significantly decreased following treatment with miR‐223‐5p mimics compared to the NC group (Figure 5P,Q; Figure S6B, Supporting Information). We also transfected Pan02 cells with a miR‐223‐5p inhibitor and a negative control (in‐NC). The expression level of miR‐223‐5p in inhibitor‐EVs was significantly reduced compared to in‐NC‐EVs (Figure S6C, Supporting Information). After incubating C2C12 myotubes separately with in‐NC‐EVs and inhibitor‐EVs, the results showed that the diameter of myotubes treated with inhibitor‐EVs was significantly larger than those treated with in‐NC‐EVs (Figure S6D,E, Supporting Information). These findings suggest that targeting miR‐223‐5p may offer a promising strategy to mitigate muscle wasting in pancreatic cancer.

### 2.6 miR‐223‐5p in EVs Induces Muscle Wasting by Targeting MAFA

**Figure 6:**
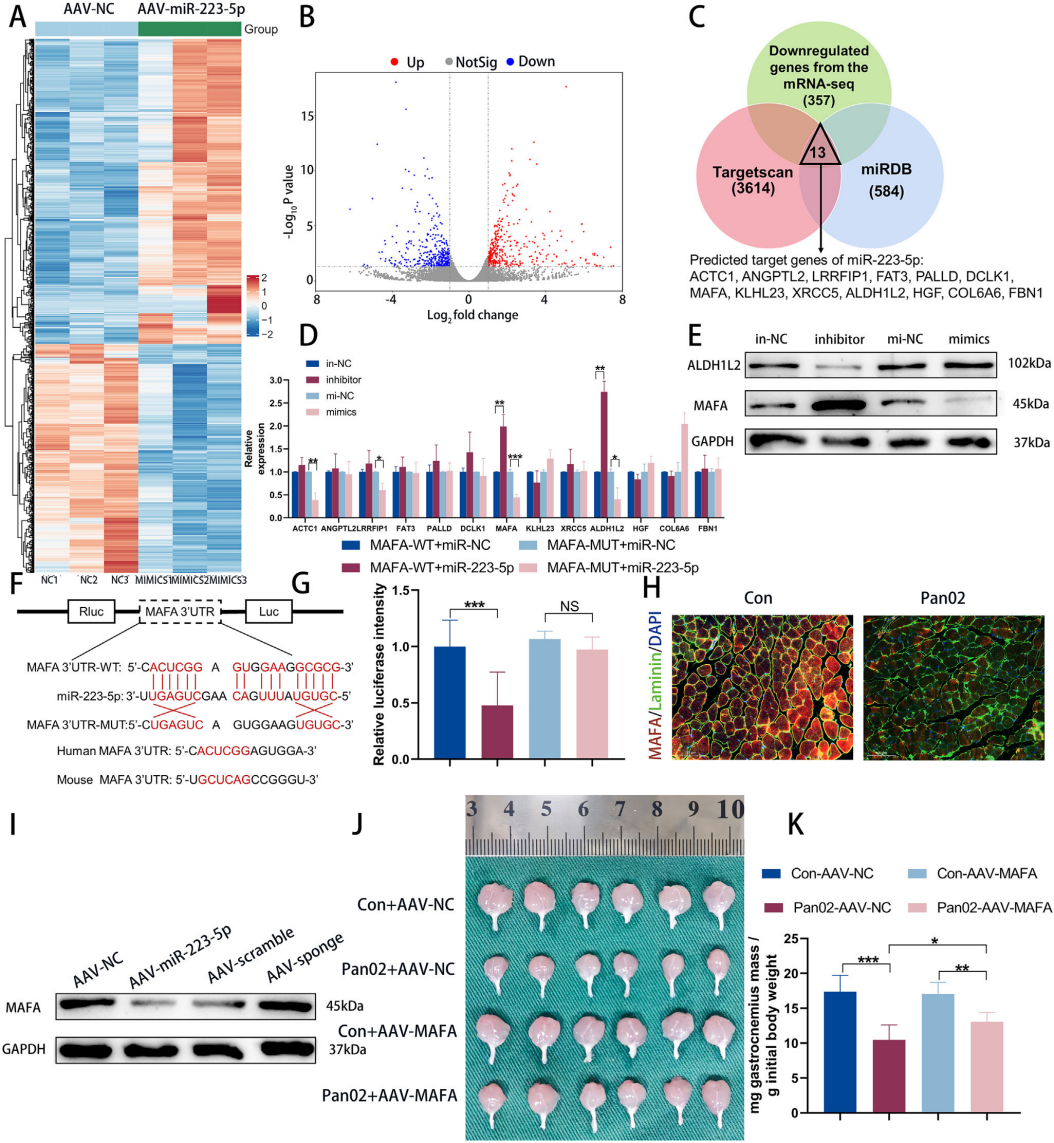
miR‐223‐5p in EVs induces muscle wasting by targeting MAFA. A) The heatmaps of mRNA sequencing showed the differentially expressed genes in muscles treated with AAV‐miR‐223‐5p or AAV‐NC. B) The volcano map of mRNA sequencing showed the differentially expressed genes in muscles treated with AAV‐miR‐223‐5p or AAV‐NC. C) Venn diagram detailing the exploration of downstream genes of miR‐223‐5p from TargetScan and miRDB, together with the downregulated genes from mRNA sequencing after miR‐223‐5p overexpression (fold change > 2, P < 0.05). D) The expression of ACTC1, ANGPTL2, LRRFIP1, FAT3, PALLD, DCLK1, MAFA, KLHL23, XRCC5, ALDH1L2, HGF, COL6A6, and FBN1 after miR‐223‐5p alteration in C2C12 cells were measured by qRT‐PCR (n = 3). E) The expression of ALDH1L2 and MAFA after miR‐223‐5p alteration in C2C12 cells was measured by Western blot. F) Schematic diagram of the dual luciferase reporter vector containing the wild‐type or mutant sequences of the 3′‐UTR of MAFA. G) Relative fluorescence intensity of dual luciferase reporter assays with wild‐type or mutant‐type MAFA 3′‐UTRs performed with or without overexpression of miR‐223‐5p in C2C12 cells was quantified (n = 3). H) Representative immunofluorescent images of MAFA in muscle tissues of con and Pan02 mice. Scale bars, 100 µm. I) The expression of MAFA after miR‐223‐5p alteration in the muscles of mice was measured by Western blot. J,K) GA muscle images (J) and muscle weight analysis (K) of C57BL/6J mice after AAV injection (n = 6). Data shown as mean ± SD. ^*^ *P* < 0.05, ^**^ *P* < 0.01, ^***^ *P* < 0.001.

To gain deeper insight into the mechanisms underlying miR‐223‐5p‐mediated muscle wasting, we conducted RNA sequencing on GA muscles infected with AAV‐miR‐223‐5p (**Figure** **6**A,B). Predicted targets of miR‐223‐5p were obtained from miRDB and TargetScan, and cross‐referenced with downregulated genes from the RNA sequencing data of miR‐223‐5p‐overexpressing GA muscles versus control GA muscles (fold change > 2, adjusted P < 0.05). Thirteen genes were identified through this approach (Figure 6C). qRT‐PCR analysis revealed that overexpression of miR‐223‐5p decreased the expression of MAFA and ALDH1L2, while miR‐223‐5p inhibition increased their expression (Figure 6D). However, at the protein level, only MAFA expression was significantly upregulated with miR‐223‐5p inhibition, with no significant change in ALDH1L2 protein levels (Figure 6E). The dual luciferase reporter assay further confirmed that miR‐223‐5p directly binds to the 3′UTR of MAFA, validating MAFA as a downstream target of miR‐223‐5p (Figure 6F,G). Immunostaining showed reduced MAFA expression in the GA muscles of tumor‐bearing mice compared to control mice (Figure 6H). Additionally, miR‐223‐5p overexpression suppressed MAFA expression in GA muscles, while AAV‐miR‐223‐5p‐sponge treatment mitigated the reduction of MAFA levels in GA muscles in tumor‐bearing mice (Figure 6I). Finally, we examined whether MAFA could alleviate cachexia‐associated muscle wasting induced by pancreatic cancer. For this, AAV‐MAFA or control viral vectors were administered into the GA muscle 3 days prior to injecting Pan02 cells into the pancreas tail of mice. AAV‐MAFA‐injected tumor‐bearing mice exhibited a trend toward increased muscle weight compared to AAV‐NC‐injected tumor‐bearing mice (Figure 6J,K). These findings suggest that MAFA may serve as a protective factor against muscle wasting.

### 2.7 EV‐miR‐223‐5p Inhibits METTL14 Transcription by Targeting MAFA in Muscles

**Figure 7:**
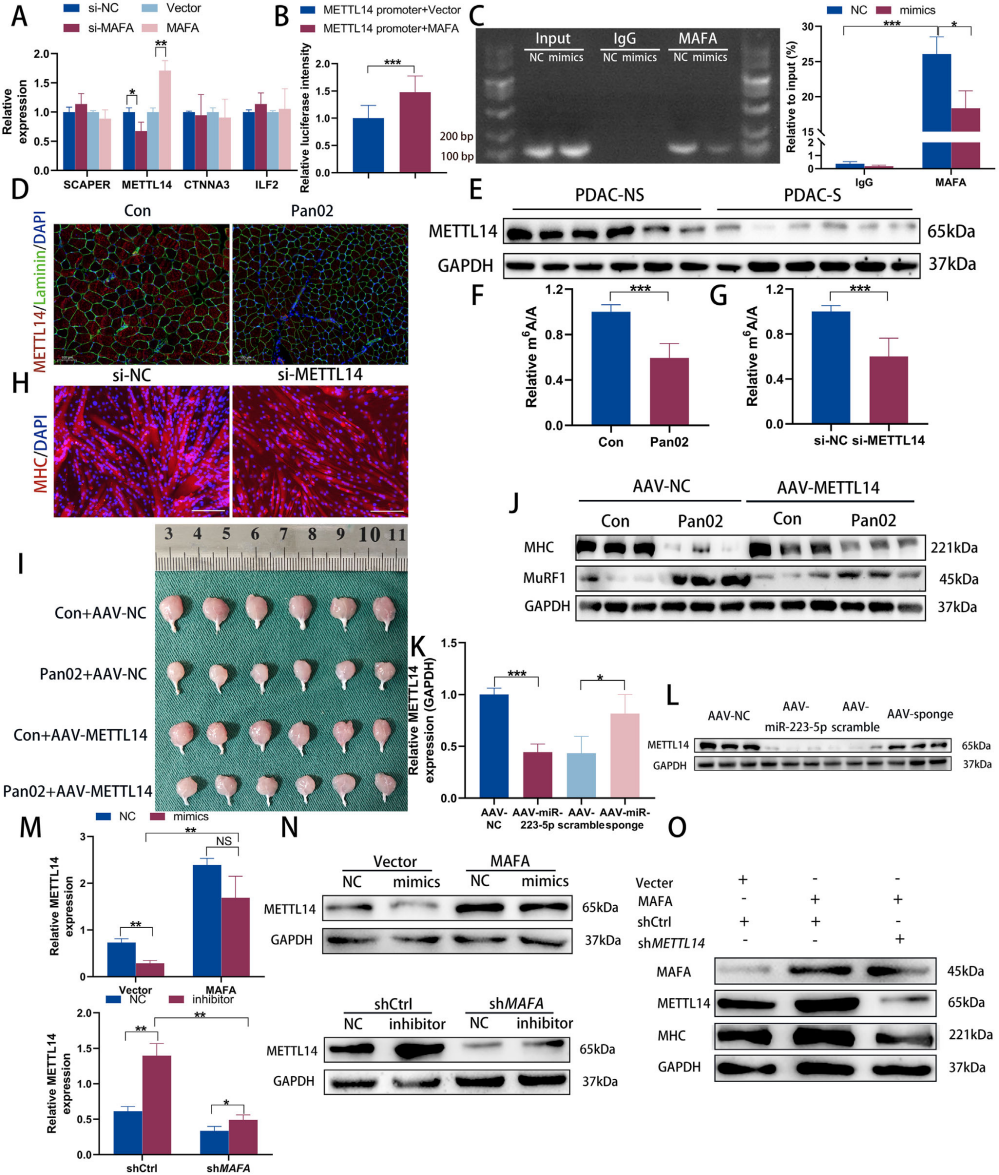
EV‐miR‐223‐5p inhibits METTL14 transcription by targeting MAFA in muscles. A) The expression of SCAPER, METTL14, CTNNA3, and ILF2 after MAFA alteration in C2C12 cells was measured by qRT‐PCR (n = 3). B) Luciferase reporter analysis of METTL14 promoter luciferase reporters in C2C12 cells transfected with MAFA or control (n = 3). C) qRT‐PCR was performed in C2C12 cells after ChIP by MAFA antibody or control IgG with or without miR‐223‐5p overexpression (n = 3). D) Representative immunofluorescent images of METTL14 in muscle tissues of con and Pan02 mice. Scale bars, 100 µm. E) Western blot of METTL14 protein in rectus abdominis muscle from non‐sarcopenia PDAC and sarcopenia PDAC patients. F,G) Quantification of m^6^A level relative to total adenosine (m^6^A/A) as determined by ELISA in muscles of con and Pan02 mice (F) (n = 6) and C2C12 myotubes after METTL14 alteration (G) (n = 3). H) The representative images of immunofluorescence staining for MHC in C2C12 myotubes after METTL14 alteration. Scale bars, 100 µm. I) GA muscle images Western blot of MHC and MuRF1 protein in GA muscle (J) of mice after AAV injection (n = 6). J) Western blot of MHC and MuRF1 protein in the GA muscle of mice after AAV injection (n = 3). K) The METTL14 mRNA level was measured in the GA muscle of C57BL/6J mice after miR‐223‐5p alteration by qRT‐PCR (n = 6). L) The METTL14 protein level was measured in the GA muscle of C57BL/6J mice after miR‐223‐5p alteration by Western blot (n = 3). M) The METTL14 mRNA expression was measured by qRT‐PCR in C2C12 transfected with the indicated vectors (n = 3). N) The METTL14 protein expression was measured by Western blot in C2C12 transfected with the indicated vectors. O) Western blot analysis of MHC after MAFA overexpression with or without sh*METTL14*. Data shown as mean ± SD. ^*^ *P* < 0.05, ^**^ *P* < 0.01, ^***^ *P* < 0.001.

MAFA, a member of the large MAF family of transcription factors (TFs), functions as a transcriptional activator by directly binding to specific DNA regions.^[^ ^28^ ^]^ To identify downstream targets of MAFA, we screened the top four predicted targets from ChIP‐Atlas (SCAPER, METTL14, CTNNA3, and ILF2). qRT‐PCR analysis showed that MAFA interference reduced, while MAFA overexpression increased, the expression of METTL14 (**Figure** **7**A). However, the expression levels of the other three genes were not significantly affected by MAFA alteration (Figure 7A). To further investigate the relationship between MAFA and the METTL14 promoter region, we conducted a luciferase reporter assay. Results showed that MAFA significantly enhanced the luminescence of the luciferase reporter containing the METTL14 promoter (Figure 7B). Additionally, ChIP‐PCR analysis in C2C12 cells confirmed the binding of MAFA to the METTL14 promoter region, an effect that could be suppressed by miR‐223‐5p overexpression (Figure 7C). These findings collectively demonstrate that MAFA directly binds to the METTL14 promoter and promotes its transcriptional expression.

METTL14, an m^6^A‐modified methylase (writer), regulates m^6^A modification levels of mRNA, one of the most abundant and well‐characterized post‐transcriptional modifications in eukaryotic mRNA.^[^ ^29^ ^]^ m^6^A methylation of mRNA has been shown to regulate muscle maintenance and growth in mice.^[^ ^30^ ^]^ To explore the role of METTL14 in muscle tissue, we examined its expression in mice and observed significant fluorescence enrichment in the GA muscles of the control group (Figure 7D). Western blot analysis further demonstrated that METTL14 expression was markedly higher in the muscles of PDAC patients without sarcopenia compared to those with sarcopenia (Figure 7E; Figure S7A, Supporting Information). To assess the role of METTL14 and m^6^A methylation in muscle, we measured m^6^A levels in mice, finding an overall reduction in m^6^A levels in the muscles of tumor‐bearing mice (Figure 7F). In cells where METTL14 was silenced, m^6^A levels, myotube diameters, and MHC protein levels were all significantly reduced (Figure 7G,H; Figure S7B—D, Supporting Information). Finally, we tested whether METTL14 could mitigate muscle wasting in the presence of pancreatic tumors. For this, we administered AAV‐METTL14 or control vectors into the GA muscle of mice, followed by Pan02 cell injections into the pancreas tail 3 days later. Assessment of the GA muscle in Pan02 mice injected with AAV‐METTL14 40 days post‐delivery showed greater volume and mass compared to Pan02 mice injected with AAV‐NC (Figure 7I; Figure S7E, Supporting Information). This increase in muscle mass was accompanied by a trend toward elevated MHC and decreased MuRF1 protein levels (Figure 7J; Figure S7F, Supporting Information). These findings suggest that METTL14 plays a crucial role in maintaining skeletal muscle in the context of PDAC.

Moreover, qRT‐PCR and Western blot analysis showed that overexpression of miR‐223‐5p significantly decreased METTL14 expression, while miR‐223‐5p inhibition had the opposite effect. These findings indicate that miR‐223‐5p regulates METTL14 expression both in vivo and in vitro (Figure 7K,L; Figure S7G—J, Supporting Information). Rescue experiments further confirmed that miR‐223‐5p modulates METTL14 expression by targeting MAFA in C2C12 cells (Figure 7M,N; Figure S7K, Supporting Information). Additionally, we observed that MAFA overexpression increased MHC protein levels, an effect that was reversed by METTL14 knockdown (Figure 7O; Figure S7L, Supporting Information). Together, these results suggest that METTL14 acts as a downstream target in the miR‐223‐5p/MAFA pathway and plays a crucial role in maintaining muscle mass in the context of cancer cachexia.

### 2.8 Correlation between MAFA, METTL14, and MuRF1 Expression and Association with OS in PDAC Patients

**Figure 8:**
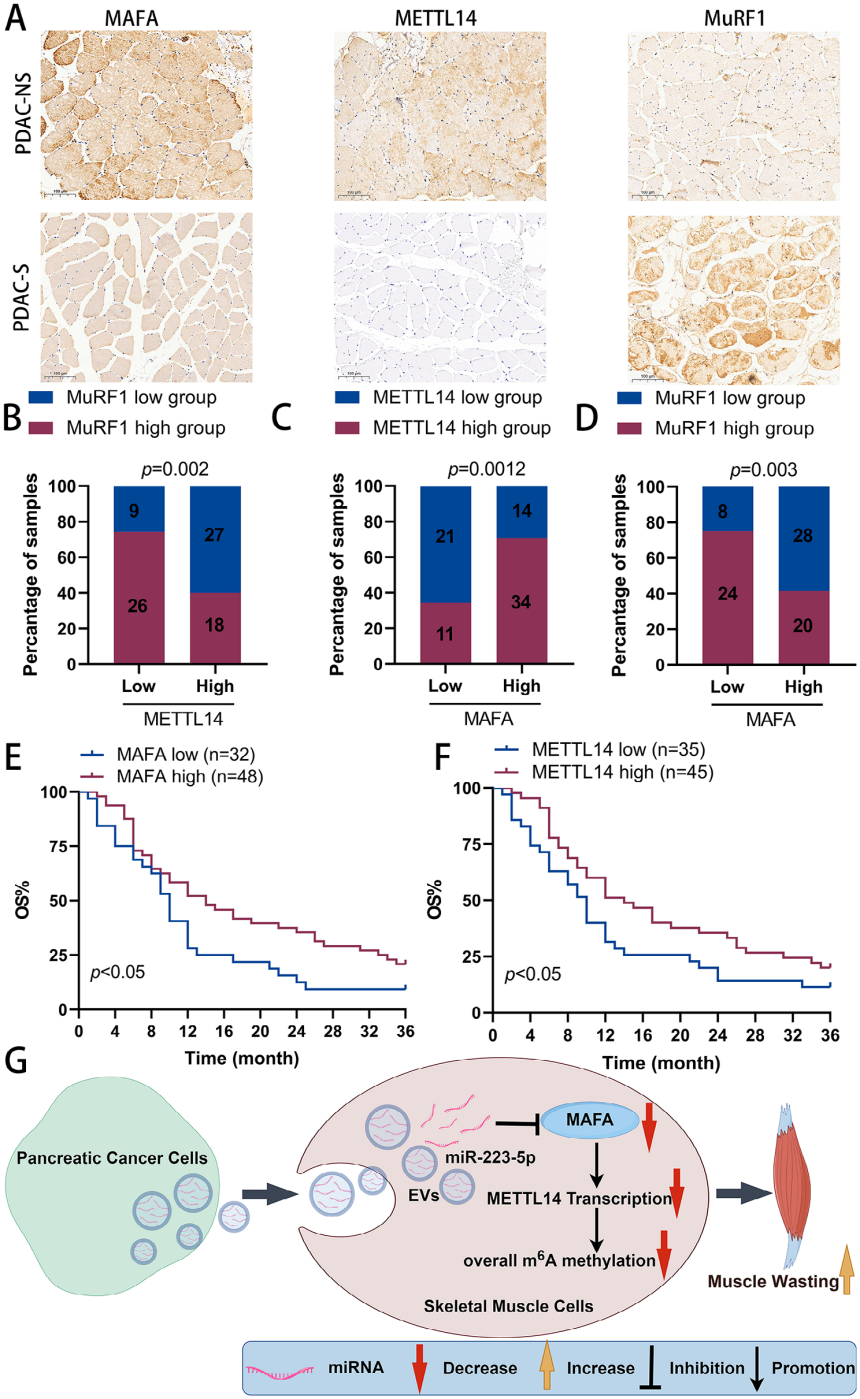
Correlation between MAFA, METTL14, and MuRF1 expression and association with OS in PDAC patients. A) Representative images of IHC staining of MAFA, METTL14, and MuRF1 of muscle samples from clinical PDAC patients. Scale bars, 100 µm. B—D) Correlation analysis of IHC data of PDAC in A. Statistical significance was determined by the chi‐square test. A total of 80 PDAC muscle specimens were analyzed. E,F) Kaplan–Meier analyses for PDAC in A. A total of 80 PDAC muscle specimens were analyzed. G) Schematic diagram of the mechanism for EVs containing miR‐223‐5p decreasing overall m^6^A levels of muscles and promoting PDAC‐associated muscle wasting by targeting MAFA and inhibiting METTL14 transcription. Data shown as mean ± SD. ^*^ *P* < 0.05, ^**^ *P* < 0.01, ^***^ *P* < 0.001.

To explore the clinical relevance of our findings, we analyzed MAFA, METTL14, and MuRF1 expression in rectus abdominis specimens from a cohort of 34 PDAC patients without sarcopenia and 46 PDAC patients with sarcopenia using immunohistochemistry (IHC). Both MAFA and METTL14 were significantly downregulated in the muscles of patients with sarcopenia (**Figure** **8**A). Additionally, our analysis revealed a positive correlation between MAFA and METTL14 protein expression, while both MAFA and METTL14 were negatively correlated with MuRF1 (Figure 8B–D). Consistent with these results, higher expression levels of MAFA and METTL14 were associated with longer OS in PDAC patients (Figure 8E,F).

## 3 Discussion

Pancreatic cancer is a malignancy with a poor prognosis and has the highest prevalence of cancer cachexia.^[^ ^31^ ^]^ Cachexia influences the quality of life and OS of individuals with pancreatic cancer.^[^ ^32^ ^]^ Unfortunately, the treatment options for managing cancer cachexia are limited.^[^ ^33^ ^]^ Therefore, identifying potential therapeutic targets to address cancer cachexia effectively remains challenging. In this study, previously unknown mechanisms of pancreatic tumor tissue‐muscle inter‐organ communication were identified. We found that EVs derived from tumor tissue and their miRNA cargo can be transferred to muscles in 2D cultured cancer cell lines, a 3D human‐derived organoid model, and an orthotopic PDAC mouse cachexia model. This transfer induces downregulation of overall m^6^A levels and muscle wasting in PDAC‐associated cachexia. Additionally, we have shown that pancreatic tumor‐derived EV miR‐223‐5p may inhibit METTL14‐mediated m^6^A methylation via the MAFA transcription factor, thereby causing PDAC‐induced muscle wasting. These findings strongly suggest a potential therapeutic strategy for ameliorating pancreatic cancer cachexia and improving patient outcomes.

EVs, which are referred to as the membrane‐bound vesicles with diameters ranging from 30 to 200 nm, are secreted by the majority of endocytic cells. Studies indicate that EVs can act as modulators in interorgan communication, including crosstalk between tumor tissue and the liver, adipose tissue and the liver, as well as adipose tissue and the brain, thus contributing to the progression of cancer metastasis, nonalcoholic fatty liver disease, and diabetes‐related cognitive impairment.^[^ ^13^, ^34^ ^]^ In the last decade, EVs in cachexia have received much attention owing to their functions in intercellular communication and tumor progression.^[^ ^35^ ^]^ Studies demonstrate that EVs played a pivotal role in developing cancer‐associated muscle wasting.^[^ ^36^ ^]^ For instance, HSP70/90‐containing EVs promote muscle wasting by activating the ubiquitin pathway in PDAC.^[^ ^37^ ^]^ Moreover, Gao et al. proposed that esophageal cancer‐EVs enriched with P4HB evoked muscle wasting by activating the PHGDH/Bcl‐2/caspase‐3 pathway.^[^ ^19^ ^]^ It remains unclear whether these EVs extracted from blood originate from tumors or other tissues, such as the liver or adipose tissue. Therefore, exploration and confirmation of the primary source of pathogenic EVs are necessary. Our results demonstrated that EVs derived from pancreatic tumor tissues can be transferred to muscles and contribute substantially to the development of muscle wasting. By treating mice and C2C12 myotubes with EVs extracted from patients with PDAC, tumor‐bearing mice, human‐derived PDAC organoids, and Pan02 cells, we found that EVs derived from tumor tissues had the capacity to trigger muscle wasting in vitro and in vivo. Moreover, administering AAV‐palmmCherry in pancreatic tumors of mice to label EVs clearly revealed that EVs secreted by tumor cells could be absorbed by myotubes, authenticating the pivotal role of EVs in the crosstalk between tumor and muscle.

EVs carry a diverse range of cargo components, such as miRNAs, other RNA species, lipids, and proteins.^[^ ^38^ ^]^ Among these, miRNAs, which are single‐stranded non‐coding RNAs composed of 19–22 nucleotides, are identified as key regulators of EV function.^[^ ^39^ ^]^ miRNAs can be encapsulated within EVs, released into circulation, and facilitate inter‐organ communication by altering gene expression in distant cells, especially in cancer.^[^ ^40^ ^]^ The high abundance, relative stability, and evolutionary conservation among species also endow miRNAs as valuable biomarkers for cancers.^[^ ^41^ ^]^ Therefore, we further explored key PDAC‐derived EV‐miRNAs involved in cachexia‐associated muscle wasting. In this study, we demonstrate that miRNAs contained within EVs derived from tumors play a significant role in contributing to the pool of circulating miRNAs.^[^ ^42^ ^]^ These tumor‐derived miRNAs can be transferred to skeletal muscles, leading to muscle mass loss and strength impairment both in patients with PDAC and in pancreatic tumor‐bearing mice. miRNA sequencing demonstrated that miR‐223‐5p was highly enriched in pancreatic tumors, EVs in plasma, and skeletal muscles of PDAC patients with sarcopenia, as well as in Pan02 mice, suggesting its potential role as a key regulator of muscle dysfunction and wasting. Furthermore, ROC curve analysis suggested that the expression levels of EV‐miR‐223‐5p in patient plasma and miR‐223‐5p in PDAC tissues had the potential to serve as biomarkers of sarcopenia. Higher miR‐223‐5p expression in the plasma also predicted poor OS in patients with PDAC. Notably, several previous studies suggest that miR‐223 is associated with cancer cachexia. For example, He et al. reported that the expression levels of miR‐223 were increased in patients with PDAC.^[^ ^43^ ^]^ Moreover, in HPV16‐transgenic mice, miR‐223‐3p played a role in muscle wasting, possibly by regulating the MAPK cascades.^[^ ^20^ ^]^ The consistencies across the published studies and ours collectively validate the significance of miR‐223 in cachexia.

Furthermore, we studied the effects and underlying mechanisms of miR‐223‐5p on muscle wasting. Our results suggest that the overexpression of miR‐223‐5p in GA muscles leads to muscle mass loss and grip strength impairment. Additionally, we conducted a transcriptome analysis to investigate the role of miR‐223‐5p in muscles. Bioinformatics analysis, coupled with subsequent experimental validation, indicated that EV‐miR‐223‐5p specifically targets MAFA in C2C12 cells, thereby diminishing MAFA expression. MAFA is a transcriptional activator that directly binds to the METTL14 promoter region, further upregulating METTL14 expression. METTL14 belongs to a large and conserved family of methyltransferases and plays a significant regulatory role in m^6^A modification.^[^ ^44^ ^]^ Previous studies report that the m^6^A modification in eukaryotic RNA regulates physiological homeostasis and disease development.^[^ ^45^ ^]^ In skeletal muscles, a robust and intricate relationship between m^6^A modification and myogenesis has been documented.^[^ ^46^ ^]^ In a previous study, m^6^A methyltransferase METTL3 and m^6^A modification have been found strongly necessary for the maintenance of muscle mass and function. Notably, METTL3‐mediated m^6^A methylation has been shown to be essential for the induction of skeletal muscle hypertrophy.^[^ ^30^ ^]^ Specifically, elevated METTL3 expression and concomitant increase in m^6^A levels promote skeletal muscle growth during hypertrophy. In contrast, in pathological atrophy, the m^6^A demethylase ALKBH5 mediates m^6^A demethylation, aggravating muscle mass loss during denervation.^[^ ^47^ ^]^ Similarly, Liu et al. demonstrated that ALKBH5 overexpression reduced m^6^A levels and induced excess loss in muscle weight, as well as a decrease in myofiber CSA in denervation models. Furthermore, skeletal muscle is susceptible to m^6^A loss during physiological aging, characterized by reduced METTL3 expression.^[^ ^48^ ^]^ Mechanistically, METTL3 deficiency in myotubes leads to cellular senescence and apoptosis, implicating m^6^A dysregulation in age‐related muscle decline. In our study, we investigated the function of METTL14 and m^6^A modification on muscle phenotypes in patients and mice. We observed that m^6^A mRNA levels of muscle tissues were notably lower in PDAC patients with sarcopenia than in PDAC patients without sarcopenia. Moreover, overexpressed METTL14 protected mice against PDAC‐induced muscle wasting and impaired grip strength. Consistently, the knockdown of METTL14 in C2C12 myotubes reduced fiber diameters in vitro. Although studies have documented the biological and pathological roles of METTL14 in inflammation, tumor metastasis, and cardiomyopathy,^[^ ^49^, ^50^ ^]^ we, for the first time, proved the crucial function of METTL14 in controlling skeletal muscle homeostasis and wasting in adults. Thus, METTL14 treatment significantly prevents miR‐223‐5p‐induced muscle wasting.

This study had some limitations that should be addressed in future studies. First, while we demonstrated miR‐223‐5p's role using inhibitor and miRNA sponge approaches, these tools inherently achieve partial suppression and carry off‐target risks. CRISPR‐based knockout would provide definitive validation of miR‐223‐5p's necessity, though current technical challenges in targeting individual mature miRNAs limit its feasibility. Second, we focused on muscle loss induced by PDAC, which has the highest incidence of all cancers. In addition to the PDAC, many other cancers, such as breast, colorectal, and esophageal cancers, are also accompanied by ongoing muscle loss and cachexia. Further research is needed to investigate whether the function of miR‐223‐5p in EVs derived from pancreatic tumors in promoting muscle consumption by acting on METTL14 is applicable to other malignant tumors. Third, even though this study is the first to demonstrate the involvement of the METTL14‐m^6^A pathway in skeletal muscle wasting, our findings do not provide insight into the specific mRNA targets modified by METTL14 and the relevant “m^6^A readers” that regulate these m^6^A‐modifed specific mRNAs. Further studies employing m^6^A‐RIP‐sequencing and m^6^A‐RIP‐qPCR analysis are needed to identify the specific m^6^A‐modified transcripts in muscles. Such insights may pave the way for novel m^6^A‐targeted therapies to combat muscle wasting in cancer cachexia. Lastly, specific surface molecules on PDAC‐derived EVs that mediate muscle tropism were not identified. The EV surface, which comprises proteins, lipids, and glycocalyx elements, plays a pivotal role in targeting recipient cells, mediating biological interactions, and enabling selective cargo delivery.^[^ ^51^ ^]^ Proteins are like “navigators” that guide EVs to accurately locate receptor cells.^[^ ^52^ ^]^ PDAC‐derived EVs may express specific surface ligands that facilitate their preferential uptake by skeletal muscle cells. This specific recognition and uptake lead to their accumulation in the muscle tissue. Future studies will explore specific surface molecules on the surface of PDAC‐derived EVs that mediate muscle tropism. In conclusion, we have uncovered previously unknown mechanisms underlying muscle wasting in PDAC‐related cancer cachexia. Our findings indicate that EVs derived from tumors can infiltrate muscle tissues and induce muscle damage and dysfunction. Their cargo miRNAs mediate communication between tumor tissue and muscle. Mechanistically, we demonstrated that EVs‐derived miR‐223‐5p contributes to decreased overall m^6^A levels of muscles and is associated with muscle wasting by targeting MAFA and inhibiting METTL14 transcription (Figure 8G). Therefore, targeting the miR‐223‐5p/MAFA/METTL14 axis could represent a potential therapeutic strategy for treating PDAC‐induced muscle loss. While current functional evidence relies on partial suppression approaches, future studies using definitive knockout models will further validate its causal necessity. These results present new evidence for the role of EVs in cachexia‐associated muscle wasting and may offer fresh perspectives on EV‐miRNA‐based approaches for diagnosis and treatment upon rigorous validation of target specificity.

## 4 Experimental Section

### Clinical Samples

Cohort 1 included 100 patients with PDAC who underwent radical resection without receiving adjuvant chemotherapy before surgery in the Department of General Surgery. This cohort included 50 patients with sarcopenia and 50 patients without sarcopenia. In total, 100 serum samples were collected from all 100 patients with PDAC. Human PDAC tumor tissues and rectus abdominis specimens were collected from the enrolled 80 patients with PDAC, comprising 46 with sarcopenia and 34 without sarcopenia. According to the algorithm established by the Asian Working Group for Sarcopenia (AWGS), a diagnosis of sarcopenia was made when individuals display reduced muscle mass and strength. As per the AWGS 2019 criteria, low muscle mass was defined as an appendicular skeletal muscle mass (ASM) index (ASM/height^2^, SMI) of less than 7.0 kg m^−2^ for men and less than 5.7 kg m^−2^ for women. Additionally, low muscle strength was defined as a grip strength of less than 28 kg for men and less than 18 kg for women.^[^ ^53^ ^]^

Cohort 2 consisted of 40 patients without PDAC. All participants underwent pancreaticoduodenectomy due to the discovery of an abdominal mass during a routine physical examination, whose pathological results showed benign tumors. Pancreatic tissues, rectus abdominis specimens, and blood samples were collected from all patients in cohort 2.

Cohort 3 included 50 healthy control (con) individuals, from whom blood samples were obtained at the Health Examination Center.

All samples (Table S1, Supporting Information) were collected at Jinling Hospital (Affiliated Hospital of Medical School, Nanjing University) between November 2021 to October 2022. Informed consent was obtained from all participants, and this study was carried out in strict accordance with the principles outlined in the Declaration of Helsinki and received approval from the Ethics Committee of Jinling Hospital.

### Mouse Cachexia Model of Pancreatic Ductal Adenocarcinoma

To establish a mouse cachexia model of PDAC, 1 × 10^6^ Pan02 cells (50 µl) or an equivalent volume of phosphate‐buffered saline (PBS, Hyclone, USA) were injected into the tail of the pancreas of 5‐week‐old female C57BL/6J mice purchased from Nanjing Huimiaoxin Biotechnology Co. Ltd (China). Body weight and grip strength were measured every 5 days. In accordance with institutional guidelines, animals were euthanized when at least four of five signs of distress were observed (loss of mobility, kyphosis, ruffled fur, dehydration, and tremor) or when more than 20% of body weight was lost within 72 h. After euthanasia, tumors, muscles, and various other organs were quickly dissected and preserved at −80 °C for further analyses, or they were fixed in 4% formaldehyde for histological staining. All procedures involving mice were conducted according to an animal protocol approved by the Animal Care and Use Committee of Jinling Hospital.

Additionally, mice were anesthetized, and 50 µl adeno‐associated virus (AAV, 1 × 10^12^ vg) was injected into the gastrocnemius (GA) muscles. The AAV was synthesized and constructed by Genepharma Biotechnology Co., Ltd. (Shanghai, China).

### Generation of Human‐Derived Pancreatic Cancer Organoids

Primary patient‐derived PDAC organoids were generated from primary resected human PDAC surgical specimens following Tuveson’ protocol.^[^ ^54^ ^]^ Briefly, tumor tissues were minced and digested with 5 mg mL^−1^ collagenase II (Thermo Fisher, USA) and 10 µg mL^−1^ DNase1(Sigma–Aldrich, USA) in Advanced DMEM/F12 supplemented with HEPES (Thermo Fisher, USA), Glutamax (Thermo Fisher, USA), and penicillin‐streptomycin at 37 °C for 90 min. The cells were then embedded in growth factor‐reduced Matrigel (Corning, USA). Organoids were subsequently digested with TrypLE (Thermo Fisher, USA) for 15 min at 37 °C for passage and then embedded in GFR Matrigel. The complete medium was refreshed every 3 days.

### Cell Culture

The mouse PDAC cell line Pan02 and mouse C2C12 myoblasts were obtained from Shanghai Zhong Qiao Xin Zhou Biotechnology Co., Ltd, and cultured in complete Dulbecco's modified Eagle’ medium (DMEM, Gibco, USA) supplemented with 10% fetal bovine serum (FBS, WISENT, Canada) and 1% penicillin‐streptomycin (Gibco, USA) solution. Cultures were maintained in a humidified chamber at 37 °C with 5% CO2. For the myogenic differentiation experiment, C2C12 myoblasts were plated in 6‐ or 12‐well plates and cultured in DMEM containing 10% FBS. Upon confluency, the medium was replaced with DMEM containing 2% horse serum (Hyclone, USA) to induce differentiation. The medium was changed every 48 h for 4 days.

### Extracellular Vesicle Isolation and Characterization

EVs were isolated using differential ultracentrifugation. For PDAC organoids and Pan02 cells, cultures were maintained in a medium containing 10% EV‐depleted FBS, which was ultra‐centrifuged at 110 000 g for 18 h at 4 °C to remove existing EVs. After culturing, supernatants were then collected and subjected to centrifugation at 300 g for 10 min to remove any cells. Subsequently, they were centrifuged again at 2,000 g for 30 min to eliminate large vesicles. The supernatants were then centrifuged at 10 000 g for 30 min to clear cell debris, followed by centrifugation at 100 000 g for 70 min to isolate EVs. Finally, the EVs pellets were centrifuged for another 70 min at 100 000 g before being resuspended in PBS. Human blood was collected in EDTA‐anticoagulated vacuum tubes while mouse blood was obtained via intracardiac puncture into an Eppendorf tube containing trisodium citrate as an anticoagulant. Plasma was obtained by centrifuging whole blood at 1,500 g for 15 min. The plasma was diluted with an equal volume of PBS, and EVs were isolated by a series of centrifugation steps at different speeds and durations as described above. Additionally, according to the previous research, the EV pellet was collected, and the supernatant was further processed with the Total Exosome Isolation Kit (from plasma) (Invitrogen, 4 484 450) following the manufacturer's instructions.^[^ ^55^ ^]^

The protein concentration of EVs was measured by the BCA assay (Beyotime, China). Regarding EVs from culture medium of the Pan02 cells, ≈50 µg of total protein content of EVs was obtained from 40 mL of culture medium. As for the plasma of patients and mice, ≈1,000 µg of total protein content of EVs could be obtained from 1 mL of plasma. The final concentration of EVs in PBS was generally 1 µg µl^−1^ total protein. The EV samples were promptly stored at 4 °C for short‐term storage and at −80 °C for long‐term storage.

To characterize EVs, particle size distribution was conducted using nanoparticle tracking analysis (NTA). The morphology of EVs was examined through transmission electron microscopy (TEM), while the protein content of EVs was evaluated using Western blot.

### In Vivo and In Vitro Extracellular Vesicles and GW4869 Treatment

For in vivo studies, 100 µg of EV suspension (per injection) was adoptively transferred into recipient mice via tail vein injection twice weekly for 2 weeks.^[^ ^55^, ^56^ ^]^ Observation began immediately after the first EV injection (recorded as Day 0) and was extended continuously for 30 days. In vitro, EVs (at 20 µg mL^−1^ concentration) were co‐cultured with C2C12 cells for 5 days during differentiation. The culture medium containing EVs was changed every 2 days. To inhibit exosome‐enriched EVs biogenesis, mice were intraperitoneally injected with GW4869 (2 mg kg^−1^, Sigma–Aldrich, USA) 3 times a week, starting 2 weeks after Pan02 injection. This treatment continued for 2 weeks.

### PalmmCherry Labeling of Extracellular Vesicles

To generate EVs labeled with mCherry, a palmitoylation (palm) signal was genetically fused in frame to the N‐terminus of mCherry, as previously reported.^[^ ^25^ ^]^ Palmitoylation facilitates the attachment of proteins to cellular membranes by the formation of a thioester bond between the cysteine sulfhydryl groups and palmitic acid, a fatty acid. By integrating the palm sequence with mCherry, the latter could be effectively anchored to the cellular membrane. For the experiment, AAV‐palmmCherry was administered into mouse pancreatic tumors (1 × 10^12^ vg, 5 injection sites per tumor, 1 µl per site). The AAV‐palmmCherry vector was packaged and purified by Brainvta (China).

### PKH26 Labeling and Extracellular Vesicle Trafficking

PKH26 was a lipophilic fluorescent dye that stably integrates into lipid membranes, making it suitable for tracking EVs in vivo. To effectively monitor EVs trafficking, the PKH26 fluorescent dye (Yeasen, China) was utilized to label EVs. Following staining, the EVs labeled with the fluorescent dye were washed with PBS and then isolated through ultracentrifugation at 100 000 g for 70 min at 4 °C. Subsequently, the EVs labeled with PKH26 were resuspended in PBS and administered to the recipient mice via tail vein injection. The mouse muscle tissues were harvested 24 h post‐injection of the PKH26‐labeled EVs.

For the visualization of EV uptake in vitro, C2C12 cells were treated with PKH26‐labeled EVs for 12 h and fixed with 4% paraformaldehyde (PFA) for 30 min. Cell nuclei were stained with DAPI (Sigma, USA). The incorporation of EVs into C2C12 cells was observed using a laser confocal microscope (Zeiss LSM900).

### Assessment of Grip Strength

Skeletal muscle strength in mice was assessed using a grip strength test. The forelimb grip strength of each limb was measured with a digital grip strength meter (Calvin Biotechnology Co., China). Mice were held by the tail and allowed to grasp the grid with their forepaws or all four paws. The mice were gently pulled by their tails until they released their grip. The maximum force exerted before releasing the grid, and from at least three repetitions, was considered the grip strength of each mouse.

### EchoMRI Imaging

An EchoMRI (magnetic resonance imaging) Body Composition Analyzer (Echo Medical Systems) was used for measuring longitudinal lean and fat mass as previously described.^[^ ^57^ ^]^ All body composition inferences/analyses were performed using the accompanying EchoMRI software.

### Dual‐Energy X‐ray Absorptiometry (DXA)

The body composition of mice was measured using dual‐energy X‐ray absorptiometry (Hologic, USA).^[^ ^58^ ^]^

### Transmission Electron Microscopy (TEM)

For electron microscopy, EVs were fixed with 2.5% glutaraldehyde overnight and then contrast stained in a mixture of 4% uranyl acetate and 2% methyl cellulose for 10 min at room temperature. The prepared samples were observed at 80 kV with a JEOL‐1200EX electron microscope and photographed.

Biopsy specimens were obtained at the beginning of open abdominal surgery under general anesthesia. The edge of the rectus abdominis was exposed, and a 1 cm^3^ muscle specimen was removed using sharp dissection. The specimen was cleaned of gross blood contamination with PBS. A 2 mm^3^ biopsy specimen was fixed in 2.5% glutaraldehyde for electron microscopy.

### Coculture Assay

For the coculture experiment, pancreas tissues from patients or mice with PDAC were cultured in a transwell system placed above the C2C12 cells. The culture medium from human‐derived organoids and Pan02 cells was added to culture medium of C2C12 cells. No direct cell‐cell contact between C2C12 cells and pancreas tissues or Pan02 cells was observed.

### MicroRNA Sequencing and Analysis

The EVs were isolated from 10 ml of plasma collected from 10 patients with PDAC and 10 healthy control individuals, as mentioned above. In total, 50 ng RNA of EVs per sample was used for small RNA library preparation, and miRNA sequencing was conducted on an Illumina platform (NEB, USA) at Biotree (China). Briefly, 50 ng of purified RNA was ligated to 3′ and 5′ adapters, converted to cDNA, and amplified using Illumina primers that contained unique indexes for each sample. The samples were analyzed using the llumina HiSeq 2500 platform, and raw data were generated. After the initial quality check, sequences with lengths < 16 were discarded. The adaptor‐trimmed reads were formatted into a non‐redundant FASTQ file. The miRbase 21 database served as the reference genome for human miRNA sequences. The raw counts were analyzed using the edgeR package (Bioconductor software) after TPM normalization.

### Western Blot

Protein was extracted from tissues and cultured cells using RIPA lysis buffer (Thermo Scientific, USA) supplemented with protease and phosphatase inhibitors. The protein concentration was quantified with a BCA protein assay kit (Thermo Scientific, USA). For analysis, the protein lysates were loaded onto SDS/PAGE gels, followed by a transfer to polyvinylidene difluoride membranes (Millipore, USA). The membranes were blocked with 5% nonfat milk and then probed with various primary antibodies. The membranes were then incubated with horseradish peroxidase‐conjugated secondary antibodies and were visualized using an ECL Western blotting Detection Kit (Thermo Scientific, USA). Protein levels were analyzed using ImageJ analysis software. The antibodies applied in this study were listed in Table S3 (Supporting Information).

### Immunofluorescence Staining

For immunofluorescence staining, fresh muscles dissected from mice were collected and immediately fixed in ice‐cold 4% PFA solution overnight. Following fixation, infiltration was conducted with a mixture of 30% sucrose phosphate buffer for 1 day. All samples were then snap‐frozen in optimum cutting temperature (OCT, Fisher Healthcare, USA) with dry ice and sectioned into 5‐µm‐thick cross‐sections using a cryostat. Immunofluorescence staining was performed as previously described with overnight hybridization using primary antibodies followed by incubation with appropriate secondary antibodies (Invitrogen, USA).^[^ ^59^ ^]^ Nuclei were counterstained with DAPI.

### RNA Isolation and Quantitative RT–PCR

Total RNA was extracted from tumor tissues, muscles, or C2C12 cells using TRIzol reagent (Invitrogen, USA) following the manufacturer's protocol. EV RNA was isolated using a miRNeasy kit (QIAGEN Sciences, German). When isolating RNA from 100 µg EVs, a spike‐in control miRNA (cel‐miR‐39‐3p) was added to serve as an extraction normalization control (miDETECT miRNA External Control, RiboBio, China). Real‐time quantitative PCR (qPCR) was performed with the ChamQ SYBR qPCR Master Mix (Vazyme, China). Table S4 (Supporting Information) shows the list of primer sequences.

### RNA‐seq Library Preparation and Sequencing

Total RNA was isolated from the GA muscles of mice using TRIzol reagent (Invitrogen, USA). Sequencing libraries were prepared using the U‐mRNAseq Library Prep Kit for Illumina, according to the manufacturer's guidelines. The quality of the libraries was then validated using an Agilent 2100 Bioanalyzer, after which they were normalized and pooled for sequencing. The libraries were pooled and sequenced on the Illumina NovaSeq 6000 platform, utilizing 150‐bp paired‐end sequencing reads. Clustering, sequencing, and data analysis were conducted by Kaitaibio (China).

### Enzyme‐Linked Immunosorbent Assay

To quantify the protein concentrations of IL‐1β in mouse tissues, an enzyme‐linked immunosorbent assay (ELISA) was performed following the manufacturer's instructions using an ELISA kit (Neobioscience, China).

### Fluorescence In Situ Hybridization

A Cy3‐labeled probe specific for miR‐223‐5p (5′‐CAACTCAGCTTGTCAAATACACG‐3′) was designed and synthesized by Servicebio (China). Following deparaffinization, tissue sections were permeabilized with 0.5% Triton X‐100 for 30 min. The slides were then incubated in a prehybridization buffer at 37 °C for 30 min. Subsequently, the Cy3‐labeled miR‐223‐5p probes were added and incubated with the slides in a hybridization solution at 37 °C overnight. The following day, nuclei were stained with DAPI, and the slides were imaged via a confocal microscope (Zeiss LSM900).

### Dual Luciferase Reporter Gene Assay

Wild‐type or mutant sequences of the 3′‐UTR of MAFA were cloned into the pmirGLO dual luciferase reporter vector (Genecreate, China). C2C12 cells were seeded in 96‐well plates at a density of 3 × 10^4^ cells well^−1^. The luciferase reporter vectors were co‐transfected with a miR‐223‐5p mimic or a control. After 48 h, luciferase activity was measured, and the results were expressed as the ratio of firefly to renilla luciferase activity using the Dual–Luciferase Reporter Assay System (Promega, USA).

### Chromatin Immunoprecipitation Assay

The chromatin immunoprecipitation (ChIP) assay was conducted by the Pierce Magnetic ChIP Kit (Thermo Fisher Scientific, USA) according to the manufacture's protocols. Briefly, C2C12 cells were crosslinked by 1% formaldehyde for 10 min, and the reaction was terminated by glycine solution once cells reached 80% confluence. The cells were then resuspended in a lysis buffer and sonicated on ice for 30 min. The cell lysates were incubated overnight at 4 °C with MAFA antibody or control IgG. 50 µl protein A/G magnetic beads were added to capture the antibody‐DNA complex. Afterward, DNA was extracted from the beads, and qPCR was performed to detect fragments of the METTL14 promoter region.

### Cell Transfections and Transductions

For miRNAs or siRNA transfection, C2C12 myoblasts were seeded into 12 or 6‐well plates and allowed to reach 70–80% confluency. Then the C2C12 myoblasts were transfected with miR‐223‐5p mimics (GenePharma, China) at 50 nmol mL^−1^, miR‐223‐5p inhibitor (GenePharma, China) at 100 nmol mL^−1^, si‐MAFA (GenePharma, China), and si‐METTL14 (GenePharma, China) at 100 nmol mL^−1^, or negative control using Lipofectamine 2000 (Invitrogen, Guangzhou, China). Additionally, short hairpin RNAs (shRNA) targeting Dicer, MAFA, and METTL14 were constructed using recombinant lentiviruses provided by GenePharma and scrambled lentiviral constructs serving as negative controls (shNC). Myotubes were incubated with lentiviral vectors at a multiplicity of infection of 100. The efficiency of transfection and transduction was confirmed using quantitative reverse transcription.

### m^6^A Quantification

RNA was extracted from skeletal muscles using Trizol and subjected to m^6^A quantification using the m^6^A RNA Methylation Quantification Kit (Colorimetric) (Abcam, USA) in biological triplicate, following previously described protocols.^[^ ^60^ ^]^

### Statistical Analysis

All statistical analyses were performed using IBM SPSS Statistics for Windows (version 25.0, IBM Corp., USA) and GraphPad Prism software (version 8.0.1). Quantitative data were presented as the mean ± standard deviation (SD) from at least three independent experiments. Statistical significance was determined using unpaired Student's *t*‐tests or one‐way ANOVA, as appropriate. Overall survival (OS) was assessed by the Kaplan–Meier method and analyzed with the log‐rank test. Statistical significance was set at *P* values < 0.05.

## Conflict of Interest

The authors declare no conflict of interest.

## Author Contributions

K.X. and R.S. contributed equally to this work as joint first authors. K.X. and X.‐Y.W. designed the study. K.X. and R.S. conducted the experiments. K.X., L.Z., X.G., X.‐B.W., and C.Z. analyzed and interpreted the data. K.X. and X.C. wrote the manuscript. X.‐Y.W. and X.C. conceptualized and supervised the study.

[Correction added on 07 July 2025, after first online publication: the original publication inadvertently omitted Kangjing Xu and Rongxi Shen to indicate their joint first author status.]

## Supporting information



Supporting Information

## Data Availability

The data that support the findings of this study are available from the corresponding author upon reasonable request.
