# Supplementary material for: Pancreatic Cancer‐Derived Extracellular Vesicles Enriched with miR‐223‐5p Promote Skeletal Muscle Wasting Associated with Cachexia
Source: Adv Sci (Weinh). 2025 Jul 2;12(37):e04064. doi: 10.1002/advs.202504064 (PMC12499426; doi:10.1002/advs.202504064)
Supplement: Supplementary file 1 — Supporting Information [file ADVS-12-e04064-s001.docx]

**Supplemental Figures**

**
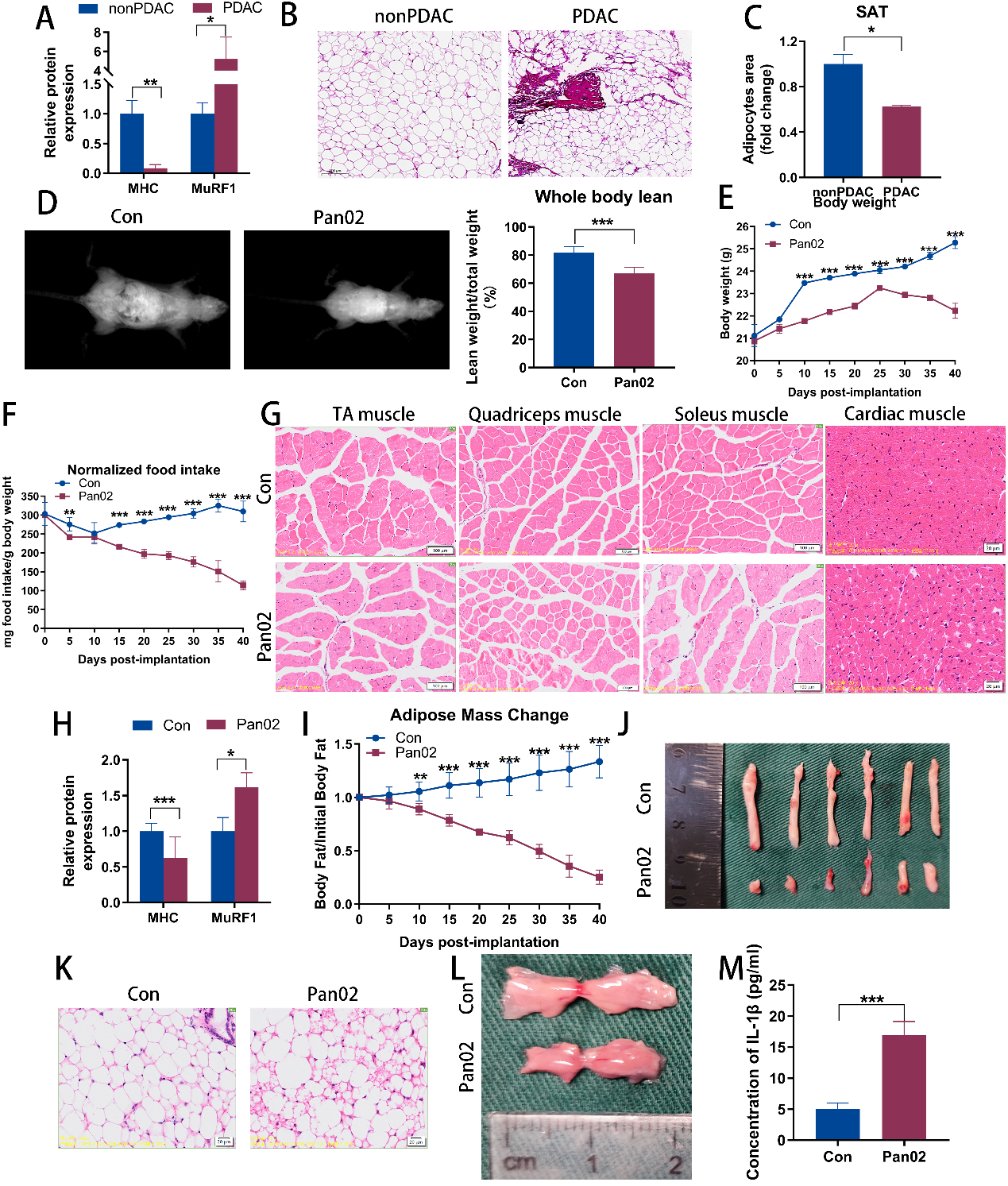
**

**Figure S1 (A)** Relative protein levels of MHC and MuRF1 in Figure 1D (n = 6). **(B)** HE staining of subcutaneous adipose tissue (SAT) from nonPDAC and PDAC patients. Scale bars, 100 μm. **(C)** Quantification of the areas of adipocytes in Figure S1B (n = 6). **(D)** Left: Lean map of tumor-bearing mice (Pan02) and non-tumor-bearing control (Con) mice by DXA. Right: Quantification of lean mass measured by DXA machine. **(E-F)** Body weight (E) and food intake (F) changes of tumor-bearing mice (Pan02, n = 6) and non-tumor-bearing control (Con, n = 6) mice. **(G)** HE staining of TA muscles, quadriceps muscles, soleus muscles (Scale bars, 100 μm) and cardiac muscles (Scale bars, 20 μm) from tumor-bearing mice and non-tumor-bearing control mice. **(H)** Relative protein levels of MHC and MuRF1 in Figure 1L (n = 6). **(I)** Adipose mass changes of tumor-bearing mice (Pan02, n = 6) and non-tumor-bearing control (Con, n = 6) mice measured by EchoMRI. **(J)** White adipose tissue (WAT) images of tumor-bearing mice (Pan02) and non-tumor-bearing control (Con) mice. **(K)** HE staining of WAT in Figure S1J. Scale bars, 20 μm. **(L-M)** Brown adipose tissue (BAT) images (L) and ELISA analysis of hippocampal IL-1β (M) of tumor-bearing mice (Pan02, n = 6) and non-tumor-bearing control (Con, n = 6) mice. Data shown as mean ± SD. *P < 0.05, **P < 0.01, ***P < 0.001.


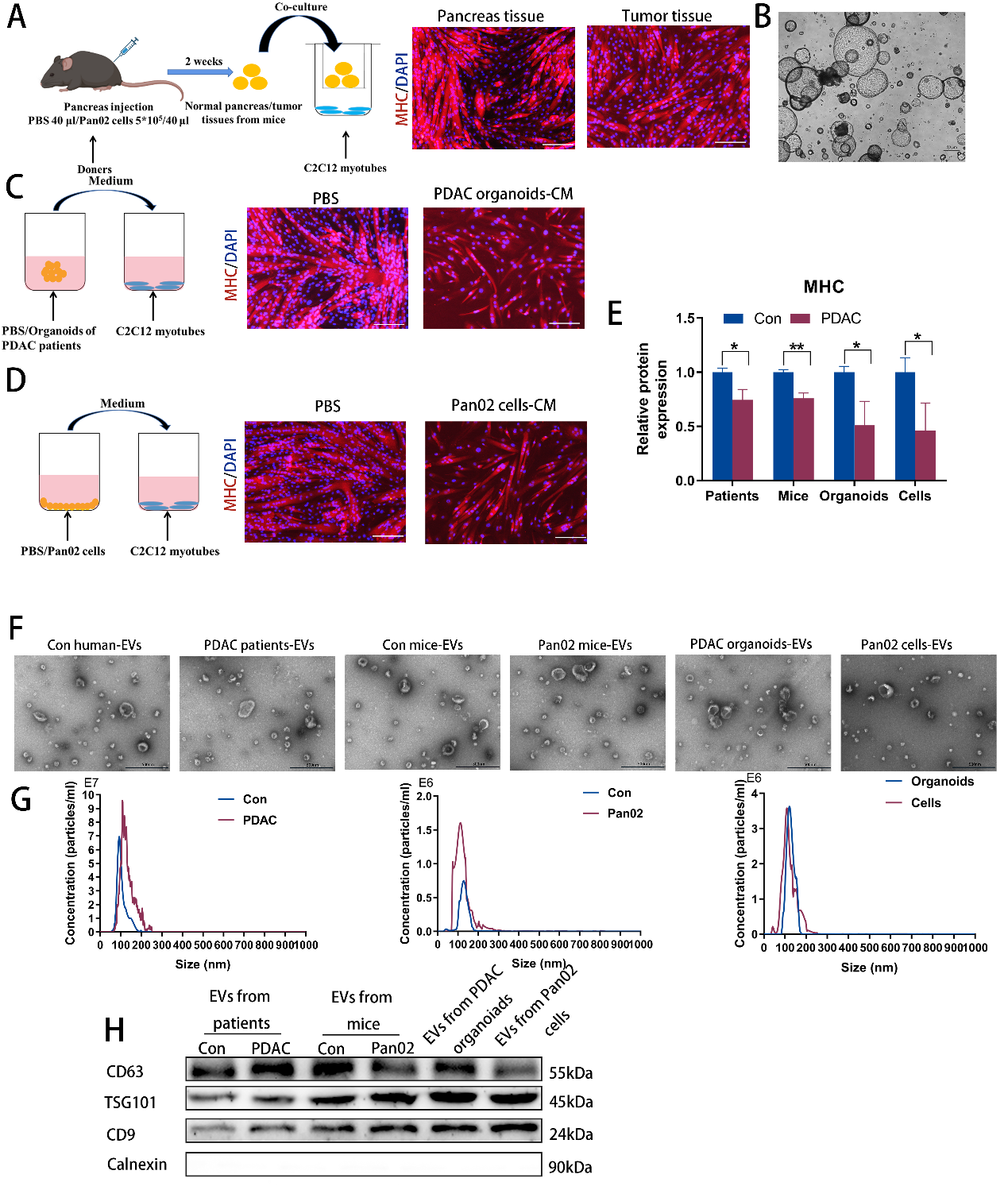


**Figure S2 (A)** Left: Experimental scheme: normal pancreas tissues of Con mice and pancreatic tumor tissues of Pan02 mice were collected in sterile procedures and then used to coculture with C2C12 myoblasts. Right: The representative images of immunofluorescence staining for MHC in C2C12 myoblasts. Scale bars, 100 μm. **(B)** Representative images of pancreatic tumor organoids. Scale bars, 50 μm. **(C)** Left: Experimental scheme: C2C12 myoblasts were treated with PBS or culture medium (CM) of human-derived PDAC organoids. Right: The representative images of immunofluorescence staining for MHC in C2C12 myoblasts treated with PBS or CM of human-derived PDAC organoids. Scale bars, 100 μm. **(D)** Left: Experimental scheme: C2C12 myoblasts were treated with PBS or CM of Pan02 cells. Right: The representative images of immunofluorescence staining for MHC in C2C12 myoblasts treated with PBS or CM of Pan02 cells. Scale bars, 100 μm. **(E)** Relative protein levels of MHC in Figure 2C (n = 3). **(F)** Microstructure of EVs from the plasma of Con and PDAC patients, Con and Pan02 mice, CM of PDAC-derived organoids, and Pan02 cells, determined by TEM. Scale bars, 500 nm. **(G)** Data of NTA analyses of EVs from the plasma of Con and PDAC patients, Con and Pan02 mice, CM of PDAC-derived organoids, and Pan02 cells. **(H)** The specific markers in EVs from the plasma of Con and PDAC patients, Con and Pan02 mice, CM of PDAC-derived organoids, and Pan02 cells detected by Western blot. Data shown as mean ± SD. *P < 0.05, **P < 0.01, ***P < 0.001.


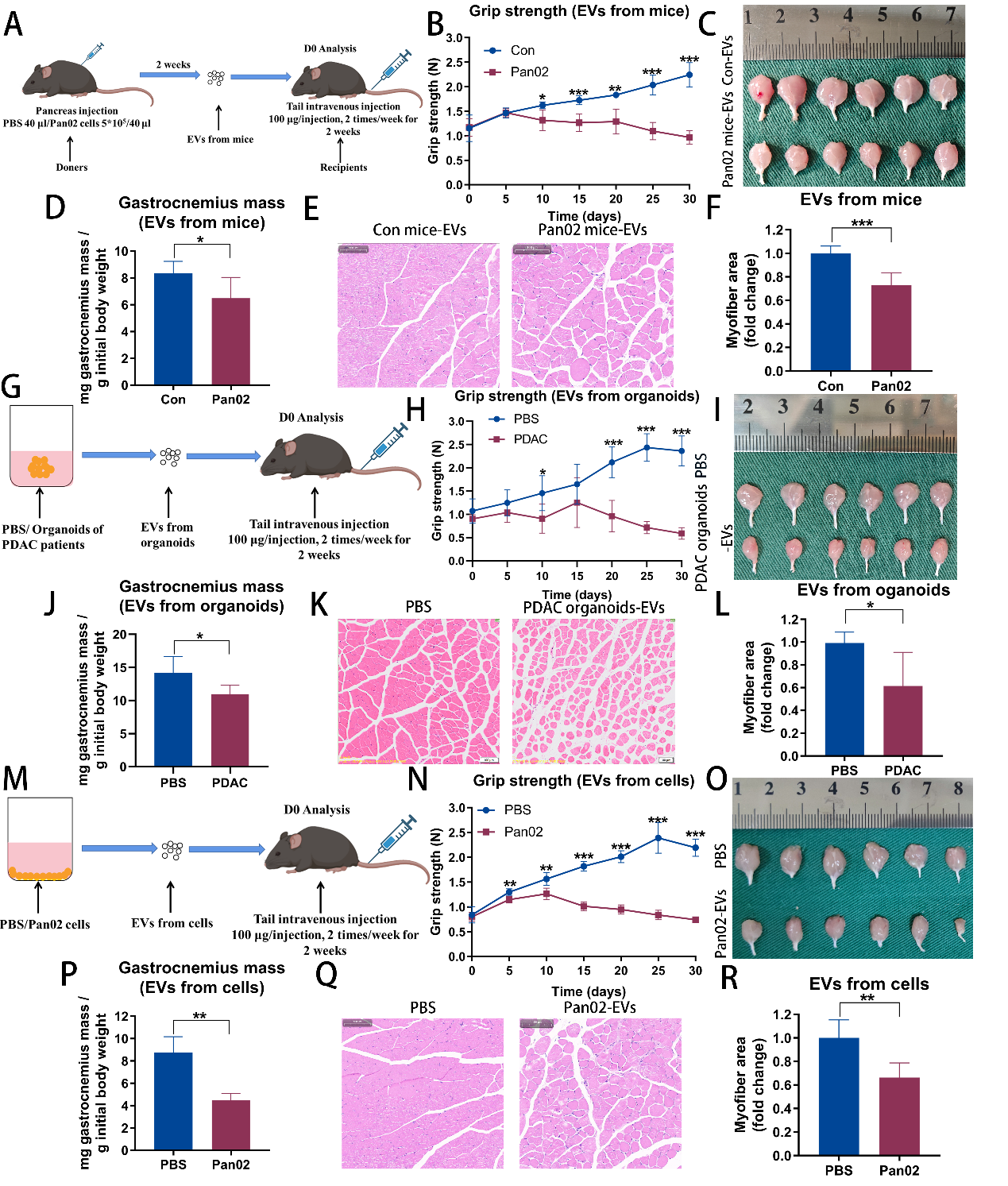


**Figure S3 (A)** Experimental scheme: EVs isolated from Con mice (Con-EVs) and Pan02 mice (Pan02 mice-EVs) were injected to mice intravenously for 2 weeks. **(B-D)** Grip strength changes (B), GA muscle images (C), and muscle weight analysis (D) of mice injected with EVs from tumor-bearing mice (Pan02, n = 6) and non-tumor-bearing control (Con, n = 6) mice. **(E)** HE staining of GA muscle from mice injected with EVs from tumor-bearing mice and non-tumor-bearing control mice. Scale bars, 100 μm. **(F)** Quantification of the myofiber CSA in Figure S3E (n = 6). **(G)** Experimental scheme: PBS or EVs isolated from CM of human-derived PDAC organoids were injected to mice intravenously for 2 weeks. **(H-J)** Grip strength changes (H), GA muscle images (I), and muscle weight analysis (J) of mice injected with PBS (Con, n = 6) or EVs from PDAC-derived organoids (PDAC, n = 6). **(K)** HE staining of GA muscle from mice injected with PBS or EVs from PDAC-derived organoids. Scale bars, 100 μm. **(L)** Quantification of the myofiber CSA in Figure S3K (n = 6). **(M)** Experimental scheme: PBS or EVs isolated from CM of Pan02 cells were injected to mice intravenously for 2 weeks. **(N-P)** Grip strength changes (N), GA muscle images (O), and muscle weight analysis (P) of mice injected with PBS (Con, n = 6) or EVs from Pan02 cells (Pan02, n = 6). **(Q)** HE staining of GA muscle from mice injected with PBS or EVs from Pan02 cells. Scale bars, 100 μm. **(R)** Quantification of the myofiber CSA in Figure S3Q (n = 6). Data shown as mean ± SD. *P < 0.05, **P < 0.01, ***P < 0.001.


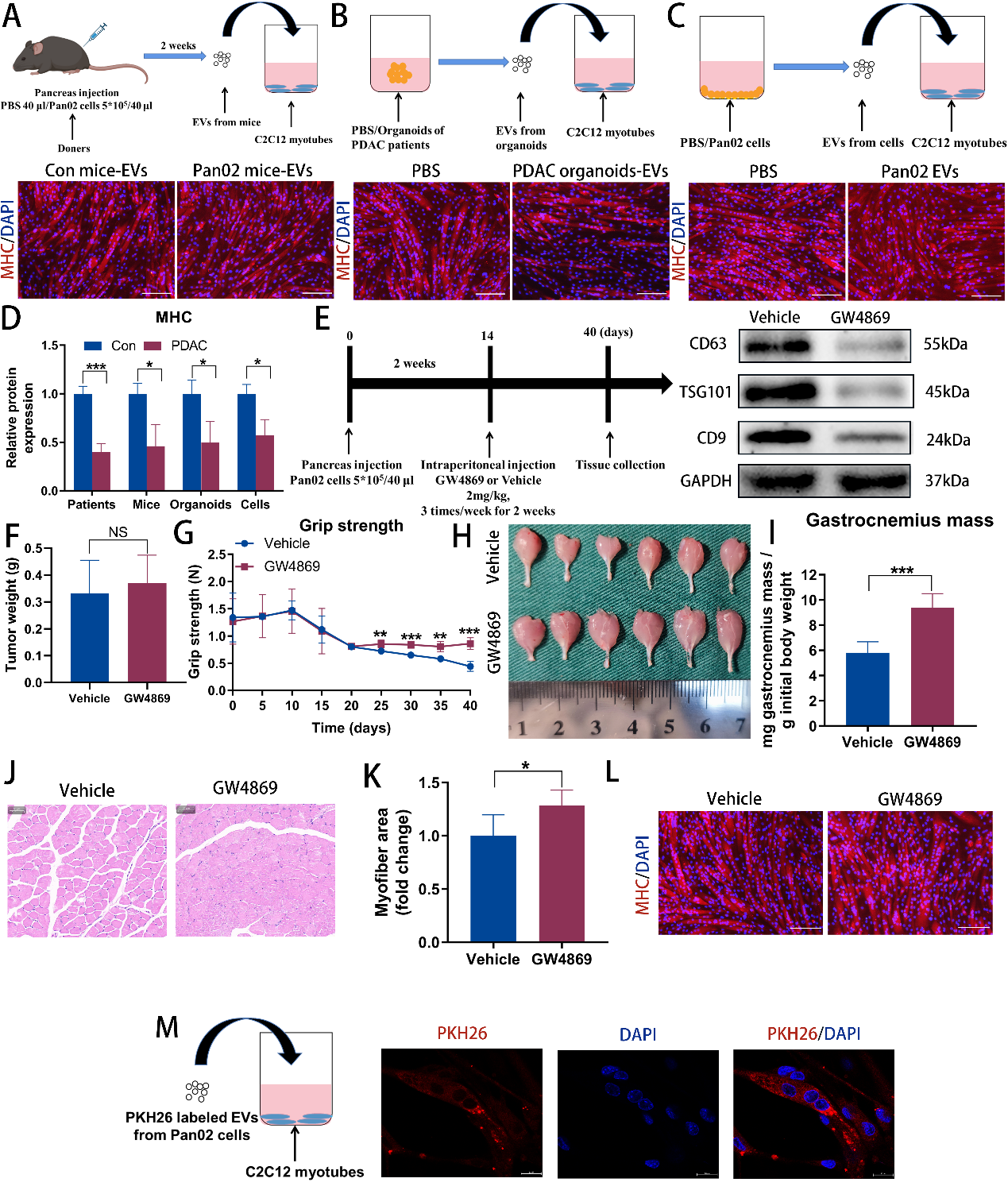


**Figure S4 (A)** Up: Experimental scheme: EVs isolated from Con mice (Con-EVs) and Pan02 mice (Pan02 mice-EVs) were used to coculture with C2C12 myoblasts. Down: The representative images of immunofluorescence staining for MHC in C2C12 myoblasts treated with EVs extracted from mice. Scale bars, 100 μm. **(B)** Up: Experimental scheme: PBS or EVs isolated from CM of human-derived PDAC organoids (PDAC organoids-EVs) were used to coculture with C2C12 myoblasts. Down: The representative images of immunofluorescence staining for MHC in C2C12 myoblasts treated with PBS or PDAC organoids-EVs. Scale bars, 100 μm. **(C)** Up: Experimental scheme: PBS or EVs isolated from Pan02 cells (Pan02-EVs) were used to coculture with C2C12 myoblasts. Down: The representative images of immunofluorescence staining for MHC in C2C12 myoblasts treated with PBS or Pan02-EVs. Scale bars, 100 μm. **(D)** Relative protein levels of MHC in Figure 2L (n = 3). **(E)** Left: Experimental scheme: GW4869 were administered intraperitoneally to Pan02 mice for a duration of 2 weeks. Right: The specific markers in EVs from the plasma of vehicle- or GW4869-treated Pan02 mice detected by Western blot. **(F-I)** Tumor weight (F), Grip strength changes (G), GA muscle images (H), and muscle weight analysis (I) of Pan02 mice treated with vehicle (Vehicle, n = 6) or GW4869 (GW4869, n = 6). **(J)** HE staining of GA muscle from Pan02 mice treated with vehicle or GW4869. Scale bars, 50 μm. **(K)** Quantification of the myofiber CSA in Figure S4J (n = 6). **(L)** The representative images of immunofluorescence staining for MHC in C2C12 myoblasts treated with EVs extracted from Pan02 cells treated with vehicle or GW4869. Scale bars, 100 μm. **(M)** Left: Experimental scheme: C2C12 myotubes were incubated with PKH26-labeled EVs. Right: Representative confocal images of PKH26 in C2C12 myotubes. Scale bars, 20 μm. Data shown as mean ± SD. *P < 0.05, **P < 0.01, ***P < 0.001.


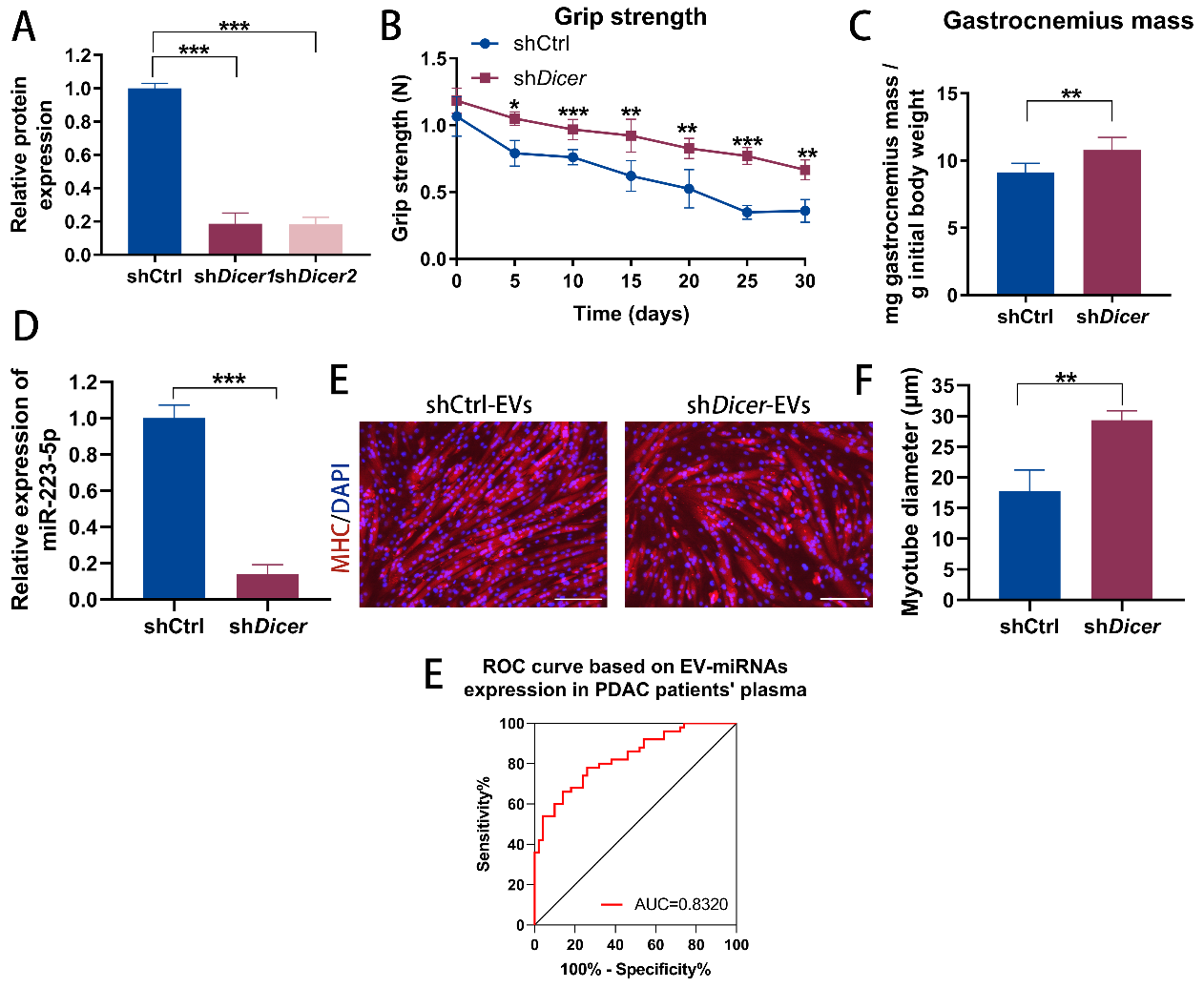


**Figure S5 (A)** Relative protein levels of Dicer in Figure 3B (n = 3). **(B-C)** Grip strength changes (B) and muscle weight analysis (C) of shCtrl (n = 6) and shDicer (n = 6) mice. **(D)** Relative levels of miR-223-5p of EVs extracted from shCtrl (shCtrl, n = 6) and shDicer (shDicer, n = 6) mice. **(E)** The representative images of immunofluorescence staining for MHC in C2C12 myoblasts treated with EVs extracted from shCtrl and sh*Dicer* Pan02 cells. Scale bars, 100 μm. **(F)** The quantitative analysis of myotube diameters of C2C12 in Figure S5E (n = 3). **(G)** ROC curves based on EV-miRNAs (miR-223-5p/378a-5p/190a-5p/148a-3p) expression in the plasma of PDAC patients. Data shown as mean ± SD. *P < 0.05, **P < 0.01, ***P < 0.001.


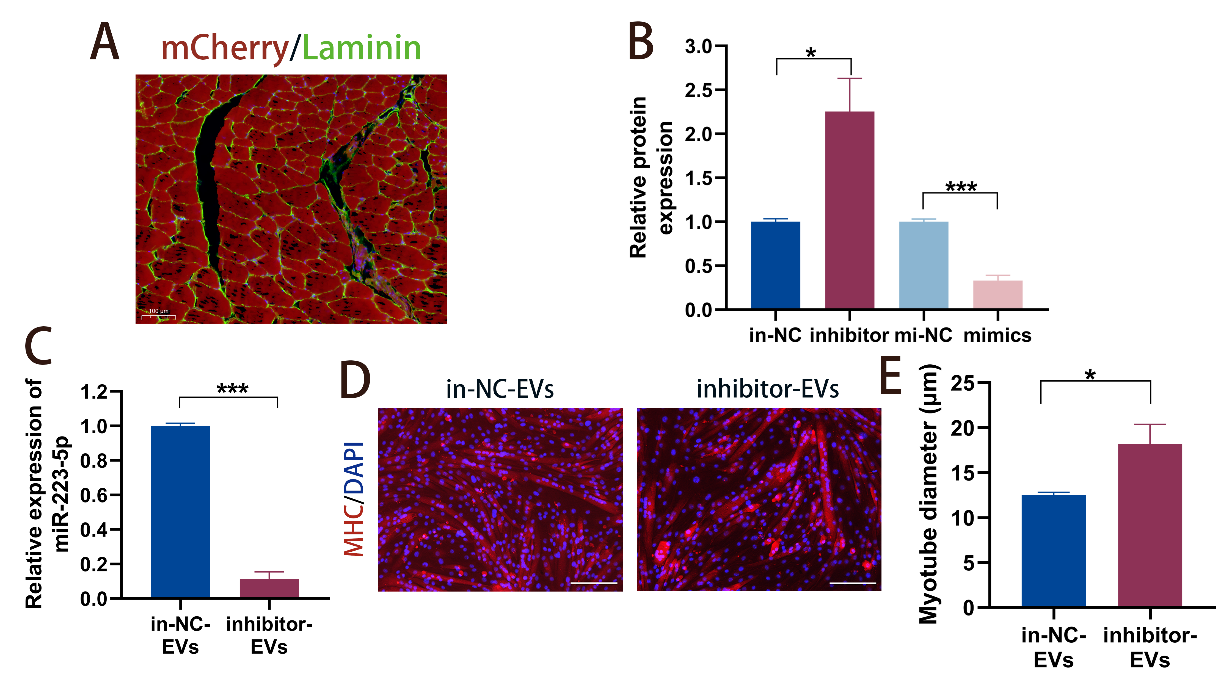


**Figure S6 (A)** Representative immunofluorescent images of mCherry and DAPI in the muscles of AAV-miR-223-5p-injected mice. Scale bars, 100 μm. **(B)** Relative protein levels of MHC in Figure 5Q (n = 3). **(C)** Relative levels of miR-223-5p of EVs extracted from Pan02 cells after inhibiting miR-223-5p expression in Pan02 cells (n = 3). **(D)** The representative images of immunofluorescence staining for MHC in C2C12 myoblasts treated with EVs extracted from in-NC and inhibitor Pan02 cells. Scale bars, 100 μm. **(E)** The quantitative analysis of myotube diameters of C2C12 in Figure S6D (n = 3). Data shown as mean ± SD. *P < 0.05, **P < 0.01, ***P < 0.001.


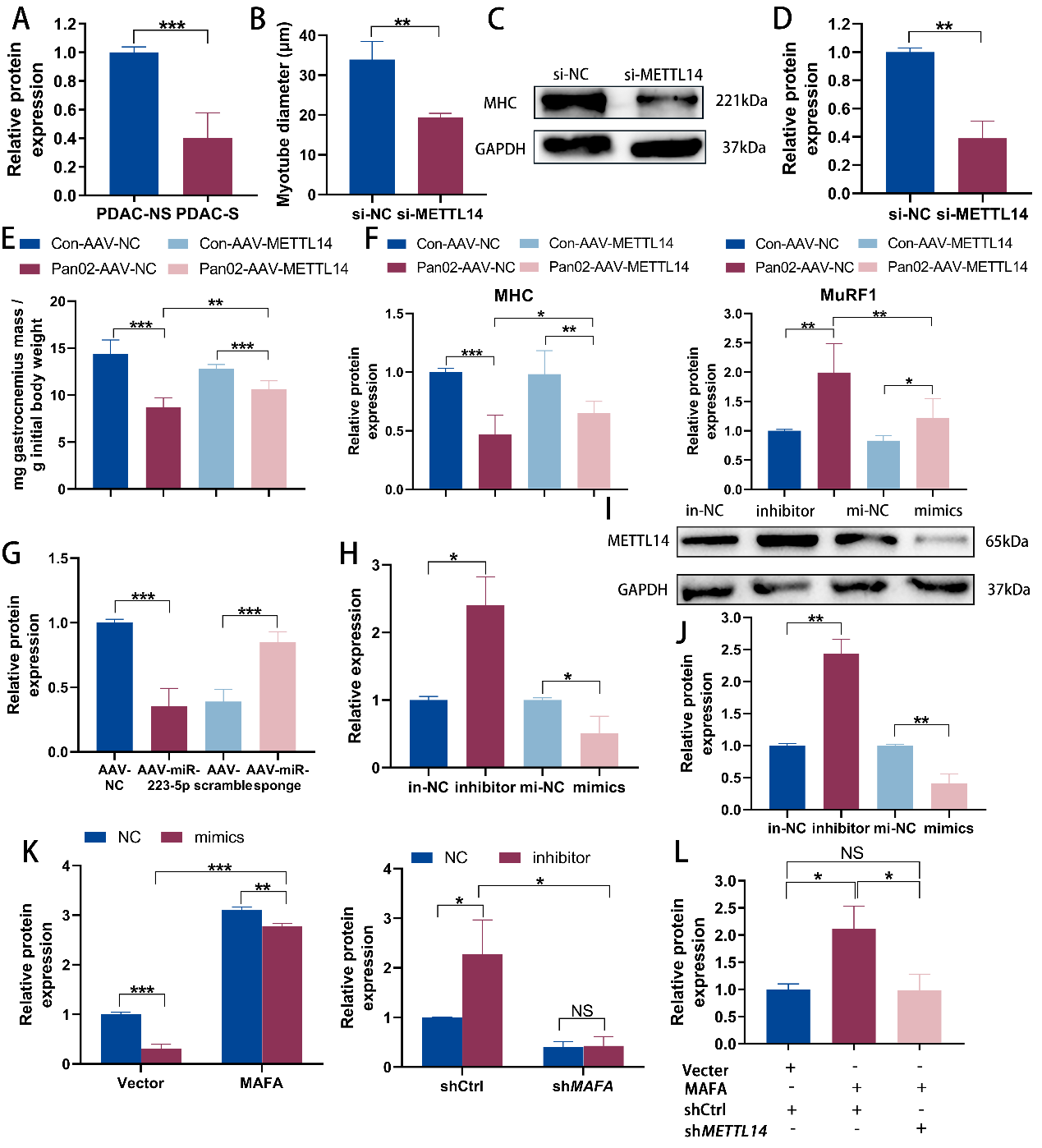


**Figure S7 (A)** Relative protein levels of METTL14 in Figure 7E (n = 6). **(B)** The quantitative analysis of myotube diameters of C2C12 in Figure 7H (n = 3). **(C)** Western blot of MHC protein in C2C12 myoblasts of Figure 7H. **(D)** Relative protein levels of MHC in Figure S7C (n = 3). **(E)** Muscle weight analysis of mice in Figure 7I (n = 6). **(F)** Relative protein levels of MHC and MuRF1 in Figure 7J (n = 3). **(G)** Relative protein levels of METTL14 in Figure 7L (n = 3). **(H-J)** qPCR analysis of METTL14 level (H), Western blot of METTL14 protein (I) and relative protein levels of METTL14 (J) after altering miR-223-5p expression in C2C12 myoblast (n = 3). **(K)** Relative protein levels of METTL14 in Figure 7N (n = 3). **(L)** Relative protein levels of MHC in Figure 7O (n = 3). Data shown as mean ± SD. *P < 0.05, **P < 0.01, ***P < 0.001.

**Table S1 Characteristics of the population**

| Characteristic | PDAC patients (N = 100) | | nonPDAC (N = 40) | Con  (N = 50) |
| --- | --- | --- | --- | --- |
|  | PDAC-NS  (N = 50) | PDAC-S  (N = 50) |  |  |
| Gender, N (%) |  |  |  |  |
| Male | 29 (58.0) | 27 (54.0) | 17 (42.5) | 22 (44.0) |
| Female | 21 (42.0) | 23 (46.0) | 23 (57.5) | 28 (56.0) |
| Age, years | 62.4 ± 11.8 | 65.7 ± 13.0 | 64.7 ± 12.5 | 61.2 ± 15.5 |
| Cohort samples, N (%) |  |  |  |  |
| Blood | 50 (100.0) | 50 (100.0) | 40 (100.0) | 50 (100.0) |
| Rectus abdominis | 34 (68.0) | 46 (92.0) | 40 (100.0) | 0 (0.0) |
| Pancreatic tissues | 34 (68.0) | 46 (92.0) | 40 (100.0) | 0 (0.0) |
| SMI, cm^3^/m^2^ | 7.6 ± 1.0 | 5.9 ± 0.7 | NA | NA |
| Male | 8.1 ± 0.7 | 6.3 ± 0.6 |  |  |
| Female | 6.9 ± 0.9 | 5.4 ± 0.6 |  |  |
| Grip strength, kg | 33.8 ± 10.3 | 18.0 ± 5.8 | NA | NA |
| Male | 37.8 ± 8.1 | 21.9 ± 4.8 |  |  |
| Female | 32.6 ± 12.8 | 13.5 ± 2.6 |  |  |

SMI, skeletal muscle index.

**Table S2 The expression of EV-miRNAs in the plasma EVs of 50 PDAC-NS and 50 PDAC-S patients.**

|  | PDAC-NS (N = 50) | PDAC-S (N = 50) | P value |
| --- | --- | --- | --- |
| hsa-miR-223-5p | 0.241 (0.127 - 0.557) | 0.521 (0.337 - 0.767) | **< 0.001** |
| hsa-miR-378a-5p | 0.274 (0.088 - 0.706) | 0.620 (0.289 - 1.178) | **< 0.001** |
| hsa-miR-190a-5p | 0.248 (0.139 - 0.389) | 0.414 (0.222 - 0.728) | **0.001** |
| hsa-miR-148a-3p | 0.412 (0.210 - 0.602) | 0.686 (0.373 - 0.996) | **0.002** |

**Table S3 Antibody list for all experiments**

| Antigen | Source | Identifier |
| --- | --- | --- |
| MHC | Proteintech | #10799-1-AP |
| MuRF1 | Proteintech | # 55456-1-AP |
| Laminin | Thermo Fisher Scientific | # PA1-16730 |
| Dicer | Proteintech | # 68375-1-lg |
| ALDH1L2 | Thermo Fisher Scientific | # PA5-120273 |
| Anti-MAFA | Boster | # A05251-1 |
| METTL14 | Cell Signaling Technology | # 48699 |
| CD63 | ABclonal | # A22343 |
| TSG101 | Proteintech | # 28283-1-AP |
| Anti-CD9 | Boster | # BM4212 |
| Calnexin | Proteintech | # 10427-2-AP |
| Anti-GAPDH | Boster | BM3876 |
| Anti-rabbit IgG-HRP antibody | Boster | # BA1054 |
| Anti-mouse IgG-HRP antibody | Boster | # BA1050 |
| Anti-Rabbit IgG, Alexa Fluor 594 conjugated | Thermo Fisher Scientific | # R37117 |
| Anti-Rabbit IgG, Alexa Fluor 488 conjugated | Thermo Fisher Scientific | # A-11034 |

**Table S4 Primer sequence for qPCR**

| Gene Name | Forward Primer | Reverse Primer |
| --- | --- | --- |
| Mus musculus |  |  |
| Pri-miR-223-5p | GGTTCCTGATCTGGCCATCT | CCTGGACCTGGAGCTGATAG |
| ACTC1 | AACTGACCCCGTCCATCAGA | TGGGTTCTGTAGGCGTGCTA |
| ANGPTL2 | CAGGAGAGAAGAGGCTTTCAG | GAACCATCCTCTGTGCCCTC |
| LRRFIP1 | TGACGGCGGAGGACGA | GTTACGGGAGCCCTTCTCAG |
| FAT3 | GTTTGACATCGTCGGGGG | TTGTTCCATCGGTGACCTCG |
| PALLD | AGCCCCTGGGAAATCCATTG | CCTGTGTCCTTGCTTTCGGT |
| DCLK1 | AGCGGAGAACCGCATTTCAA | CTTGCTTATGGAACCGGTCG |
| MAFA | GGAACGGTGATTGCTTAGGG | CGGAGGCAGAAAGAAAGACG |
| KLHL23 | TCTCCCATCATGTAAGCATGGC | GCAGCTAAAACAGCTCGGTG |
| XRCC5 | CTTGTGCACCTTCACGCTTC | CAGCACAACAGCTGCAAAGA |
| ALDH1L2 | GGGTTTTCTGTTTTCTGGGCA | GACGGCTTCTACCATGGCCT |
| HGF | TGCTCCTCCCTTCCCTACTC | CCGGGCTGAAAGAATCAAAGC |
| COL6A6 | TCAAGTGACGAACGCTATGG | ATAGAAGGACCACCTTCCCAC |
| FBN1 | TGTGGGGATGGATTCTGCTC | AGTGCCGATGTACCCTTTCTG |
| METTL14 | CTTGGGAGAGTATGCTTGCG | CCTTTGATCCCCATCAGGCA |
| GAPDH | AGGTCGGTGTGAACGGATTTG | GGGGTCGTTGATGGCAACA |
| Homo sapiens |  |  |
| Pri-miR-223-5p | TAGTTCCTGACGGTGCTGTT | TGTGCTTCTGTAGATCCGGT |
| GAPDH | CTGGGCTACACTGAGCACC | AAGTGGTCGTTGAGGGCAATG |
